# The impact of evolving mix on building’s life cycle environmental impacts under climate change: insights from a London office case study

**DOI:** 10.1007/s11367-025-02493-0

**Published:** 2025-06-13

**Authors:** Marios Kordilas, Dejan Mumovic, Yair Schwartz, Rob Cooke, Smith Mordak

**Affiliations:** 1 Institute of Environmental Design and Engineering, Bartlett School of Environment, Energy and Resources, University College London, London, UK; 2 Buro Happold Ltd, Bath, Somerset UK; 3 UK Green Building Council, London, UK

**Keywords:** Life cycle assessment (LCA), Building, Climate change, Electricity mix evolution, Beyond carbon impacts, Environmental trade-offs, Environmental burden shifting

## Abstract

**Purpose:**

The main aim of this study is to identify how evolutions in the electricity mix and climate change affect the LCA results of buildings regarding the multitude of environmental impacts. This is of critical importance now, and one that is likely to receive growing interest in the future. Firstly, because carbon might become a secondary environmental impact to mitigate as economies achieve decarbonisation milestones, and secondly, due to concerns around the trade-offs between the environmental impacts.

**Methods:**

This study evaluates the lifecycle environmental impacts of a case study office building in London by considering climate change in the UK (using CIBSE weather files) and electricity mix evolution in the UK (using National Grid ESO data), EU (using EU commission data) and China that influence operational and embodied modules of LCA. Electrification of transport is also considered, reflecting the forementioned electricity mixes. A dynamic LCA approach was followed in which the inventory was modified to reflect future electricity mixes. The influence of climate evolution was considered through dynamic thermal simulations according to London’s future climatic projections provided by CIBSE’s weather files that were then translated into lifecycle environmental impacts through the modified inventory.

**Results and discussion:**

Results of applying a dynamic approach in LCA show that there are several co-benefits of grid decarbonisation when it comes to the building’s environmental impacts. However, ecotoxicity and land occupation might come to light. Climate change led to minor reductions in the operational electricity needs, indicating that no significant savings are to be expected in the case of actively cooled buildings without free ventilative cooling. Evolving electricity mixes do not significantly reduce material embodied impacts for this case study, showing that the reduction of lifecycle impacts cannot rely only on future electricity mix evolutions. The electrification of transport was found to have an adverse effect on the building’s embodied ionising radiation impact, highlighting the importance of sourcing materials locally to avoid long transportation distances. A new type of performance gap is proposed for the building’s lifecycle environmental impacts. This can be defined as ‘the difference between the predicted and the actual environmental impact resulting from the mismatch between the actual case and the life cycle inventory’.

**Conclusions:**

Future research is needed to investigate how sensitive results are to other assumptions and how improvements in material manufacturing affect the obtained results.

## Introduction

Buildings are responsible for 37% of the global CO_2e_ emissions (United Nations Environment Programme 2021) whilst in the UK, the built environment is directly associated with 25% of the territorial CO_2e_ emissions (UK Green Building Council 2021). In 2008, the UK enacted the Climate Change Act targets, further amended in 2019 and 2023, which require a Net Zero carbon account in 2050 (The Climate Change Act 2023). Carbon budgets acting as stepping stones were set to guide the transition period: 52% of 1990 by 2027, 58% by 2032 and 78% by 2037 (Climate Change Committee 2020). Acknowledging the built environment’s role in the transition to Net Zero by 2050, the UK requires buildings to play a distinct and prominent role (HM Government 2021). It is important to recognise that during this period, climate change and electricity mixes will evolve. Climate change will influence heating and cooling demand of buildings (IEA 2023a), and electricity mixes will influence the manufacturing of most building materials.

Life cycle assessment (LCA) is an analytical and comparative framework developed by EN ISO 14040:2006 + A1:2020 document and downscaled to built environment applications by EN ISO 15978:2011 + A2:2019 and EN ISO 15804:2012 + A2:2019. These documents provide guidance for the assessment of the environmental footprint of building materials and overall projects in terms of environmental impacts. Such impacts occur throughout all phases needed to establish a building and maintain its functions, from the acquisition of raw materials to their disposal (BS EN 2021a).

All LCA impacts have an influence on United Nations (2022) climatic, water, sanitary, land and good-health Sustainable Development Goals (European Commission 2021) and include carbon and beyond carbon impacts. The first are expressed in CO_2e_, whilst under the latter’s umbrella fall all remaining impacts of EN ISO 15978:2011 + A2:2019. Yet although EN ISO 14042:2000 (the preceding document of EN ISO 14040:2006 + A1:2020) argued in 2000 that excluding temporal information diminishes the environmental relevance of impacts (EN ISO 2000), practitioners still do not consider many of those when assessing a building’s lifecycle impacts.

### Background

Dynamic LCA is an iteration of the LCA framework which considers time-varying factors. These can be climate change and electricity mix evolutions (Negishi et al. 2018), and others like technological progresses (machine efficiency, material consumption, varying energy mixes etc.), varying occupancy profiles and dynamic characterisation factors (i.e. a unit of emission released today might have a different impact than that released decades later) (Su et al. 2017). Dynamic LCA can improve the accuracy of the obtained results (Lueddeckens et al. 2020) and inform more adequately the resultant decision-making. Yet dynamic LCA is not the norm. Practitioners work with weather data files and electricity mixes only relevant for the current time (IEA 2023a); however, buildings have a lifespan in which these are expected to change. Firstly, climate change is expected to alter the operational energy demand of buildings (Jenkins et al. 2008; Chow and Levermore 2010; Kolokotroni et al. 2012; Berger et al. 2014; Walsh et al. 2015; Hooyberghs et al. 2017; Moazami et al. 2019; Larsen et al. 2020). Secondly, electricity mixes are to undergo a drastic expansion of low carbon generation to facilitate UK’s carbon budgets. Considering these, the extent to which lifecycle environmental impacts are to be influenced under dynamic conditions remains an open challenge (Roux et al. 2016; IEA 2023a).

#### Changes in electricity mix

**Fig. 1:**
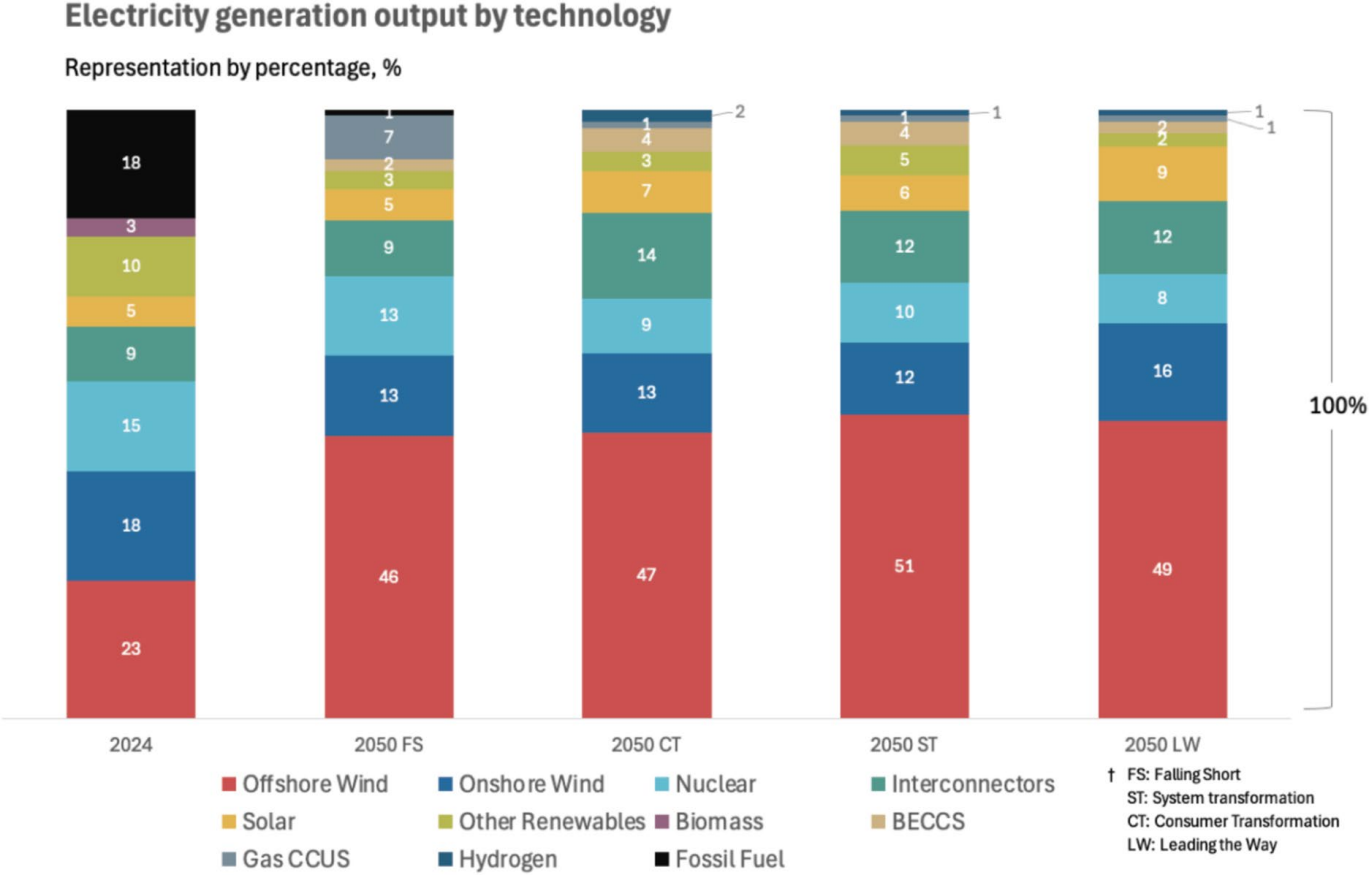
Future scenarios for UK’s electricity mix

To consider electricity mix evolution when calculating a building’s embodied and operational environmental impacts, future electricity data are needed. National Grid Electricity System Operator (ESO) (2023) provide four scenarios for UK’s future electricity compositions: Falling Short (FS), Consumer Transformation (CT), System Transformation (ST) and Leading the Way (LW). FS is assumed to be failing in delivering a Net Zero grid by 2050, whilst the three others (CT, ST, LW) deliver Net Zero by 2050 yet at different paces. All scenarios assume a significant increase in offshore wind electricity and a noteworthy increase in solar (National Grid ESO 2023) (Fig. 1).

Similar to National Grid ESO, European Commission (2020b) provides forecasts for the electricity mixes of EU countries yet without offering distinct scenarios. Across EU countries, trends show an increase in wind electricity and photovoltaics. Another source of data for future electricity compositions is International Energy Agency (IEA), IEA (2023b), which expects a global uptake of wind and solar electricity. If all forementioned projections proved true, the implications on beyond carbon impacts of buildings need investigation as these technologies are known to be associated with ecotoxicity and land-related issues, albeit being less carbon intensive (Luderer et al. 2019; UNECE 2021).

#### Climate change

Operational modules of buildings tend to be influenced by changes in the climatic context at which they operate, induced by climate change. IPCC (2022) estimates that the average surface temperature on Earth will rise 1–4.7 °C by 2100 whilst Met Office (2022) expects an increasing temperature trend in the UK. Such changes in climate will influence the need for energy—and the associated environmental impacts—so that indoor conditions can remain within comfort levels.

When estimating the impact of climate change on buildings’ operational environmental impacts, regionalised weather files are needed (Roux et al. 2016) to provide information for localised climatic inputs (e.g. dry bulb temperature, dew point, relative humidity, wind speed and direction and irradiation) (Yassaghi et al. 2020). Weather files provide hourly data useful for dynamic thermal simulation from which the energy consumption of buildings can be forecasted. CIBSE (2021a) produces weather files for several locations in the UK both for current and future climate; future files contain data under low, medium and high emission pathways. CIBSE uses its present-day climate weather files and, through morphing equations, shifts and stretches climate variables to the future (Machard et al. 2020) to produce future weather files. Another source of future weather files for the UK is Prometheus (Eames et al. 2011). Both CIBSE and Prometheus projections are of the probabilistic sort, as they represent the likelihood that the mean temperature will be less than predicted (e.g. 10 th, 50 th and 90 th), and are generated based on UKCP09 climatic information. However, CIBSE weather files are generated using three primary (i.e. air temperature, relative humidity and cloud cover) and one secondary (i.e. wind speed) variable (CIBSE 2016) whilst Prometheus uses only daily precipitation as its variable (Eames et al. 2011).

#### Electricity mix evolutions coupled with climate change

Much research has been done on carbon emissions of buildings in light of climatic and electricity evolutions. Roux et al. (2016) investigated the influence of future climatic data on operational environmental impacts in the case of low energy buildings in France’s evolving electricity mix. Their results showed significant differences across most impacts compared to static LCA. However, their analysis lacked certain dynamic dimensions, namely solar radiation, precipitation or humidity changes which, similar to heating degree days, can lead to inaccuracies (Cellura et al. 2018; Guarino et al. 2022). Further, electricity mix evolution was assumed to influence only operational impacts without regard to the embodied impacts that can be impacted by electricity evolution due to its involvement in manufacturing of materials. Another study’s findings showed similar changes for the energy demand and carbon emissions in the US (Phillips et al. 2022). Previous research (Fouquet et al. 2015; Potrč Obrecht et al. 2021) found that when evolving electricity mixes are considered, LCA leads to significantly lower carbon footprint results. This accords with similar studies in Asian cities that had shown a decreasing trend when it comes to operational carbon emissions (Su et al. 2020; Duan 2023). This decreasing trend in buildings’ carbon emissions has raised concerns around beyond carbon impacts, assuming that carbon might become a secondary environmental impact to mitigate as economies achieve decarbonisation milestones.

When it comes to beyond carbon impacts, a consensus is yet to be reached, mainly driven by the fact that not many studies explore or report beyond carbon LCA impacts. Although studies show that renewable generated electricity is associated with important beyond carbon impacts, namely ecotoxicity and land use (Luderer et al. 2019; Raugei et al. 2020; UNECE 2021; Sacchi et al. 2022), literature has yet to reach an agreement as to how these would be influenced by changes in climate and electricity when it comes to the building level. Collinge et al. (2013) found that all LCA impacts benefit from grid decarbonisation. Ayagapin et al. (2021) although reported on beyond carbon impacts for their baseline scenario, considered only carbon when it came to future electricity mixes. Ramon et al. (2023) considered the influence of climate change on the lifecycle environmental impacts of an office building in Belgium. Land use, toxicity and ionising radiation changed the most. Other studies evidenced a burden shifting of environmental impacts when transitioning from gas boilers to heat pumps in the UK and Germany (Sevindik et al. 2021; Naumann et al. 2022). Although reductions were achieved for carbon, the burden was shifted towards ecotoxicity, ozone depletion and metal depletion. Similar studies (Collinge et al. 2018; Barros et al. 2020; Ramon and Allacker 2021) found that the electricity mix chosen in LCA has a strong influence on the results of most impacts, highlighting the importance of a holistic reporting on lifecycle environmental impacts to avoid the trade-offs (Table 1).

**Table 1:** Findings from selected studies

Study	Dynamic considerations	Key findings
Collinge et al. (2013)	- Energy mix - Building use	All LCA impacts are to either reduce or increase depending on the electricity mix
Ramon et al. (2023)	- Climate change - Electricity mix	Most operational LCA impacts are sensitive to dynamic considerations with land use, toxicity and ionising radiation being the most influenced ones
Sevindik et al. (2021)	- Electricity mix - Heat pumps COP	Reductions are achieved for operational carbon at the expense of beyond carbon impacts
Naumann et al. (2022)	- Electricity mix	
Collinge et al. (2018)	- Electricity mix	Static LCA underestimates the use phase impacts of low carbon buildings
Ramon and Allacker (2021)	- Electricity mix	Static LCA overestimates carbon whilst underestimating human toxicity, particulate matter and land use
Roux et al. (2016)	- Climate change - Electricity mix	Increases are forecasted for all operational environmental impacts

In sum, even though literature agrees that carbon emissions of buildings benefit from grid decarbonisation, the future of beyond carbon impacts under dynamic considerations remains largely unknown. This information is not only important for planning future measures related to the use and replacement of building materials (Potrč Obrecht et al. 2021) but also for correctly calculating the co-benefits and trade-offs of grid decarbonisation.

### Aim of this study

This study aims to identify how evolutions in the electricity mix and climate change affect the LCA results of buildings across the multitude of environmental impacts. This is of critical importance now, and one that is likely to receive growing interest in the future. Firstly, because carbon might become a secondary environmental impact to mitigate as economies achieve decarbonisation milestones, and secondly, due to concerns around the trade-offs between the environmental impacts.

## Research methods: dynamic LCA

Although this analysis utilises aspects from commonly used approaches (EN ISO 14040:2006 + A1:2020, EN ISO 15804:2012 + A2:2019, EN ISO 15978:2011 + A2:2019 and RICS WLC guidance (BS EN 2020, 2021b, a; RICS 2023)), it goes way beyond by identifying how the building LCA results change considering climate change and varying electricity mixes.

### Research design

**Fig. 2:**
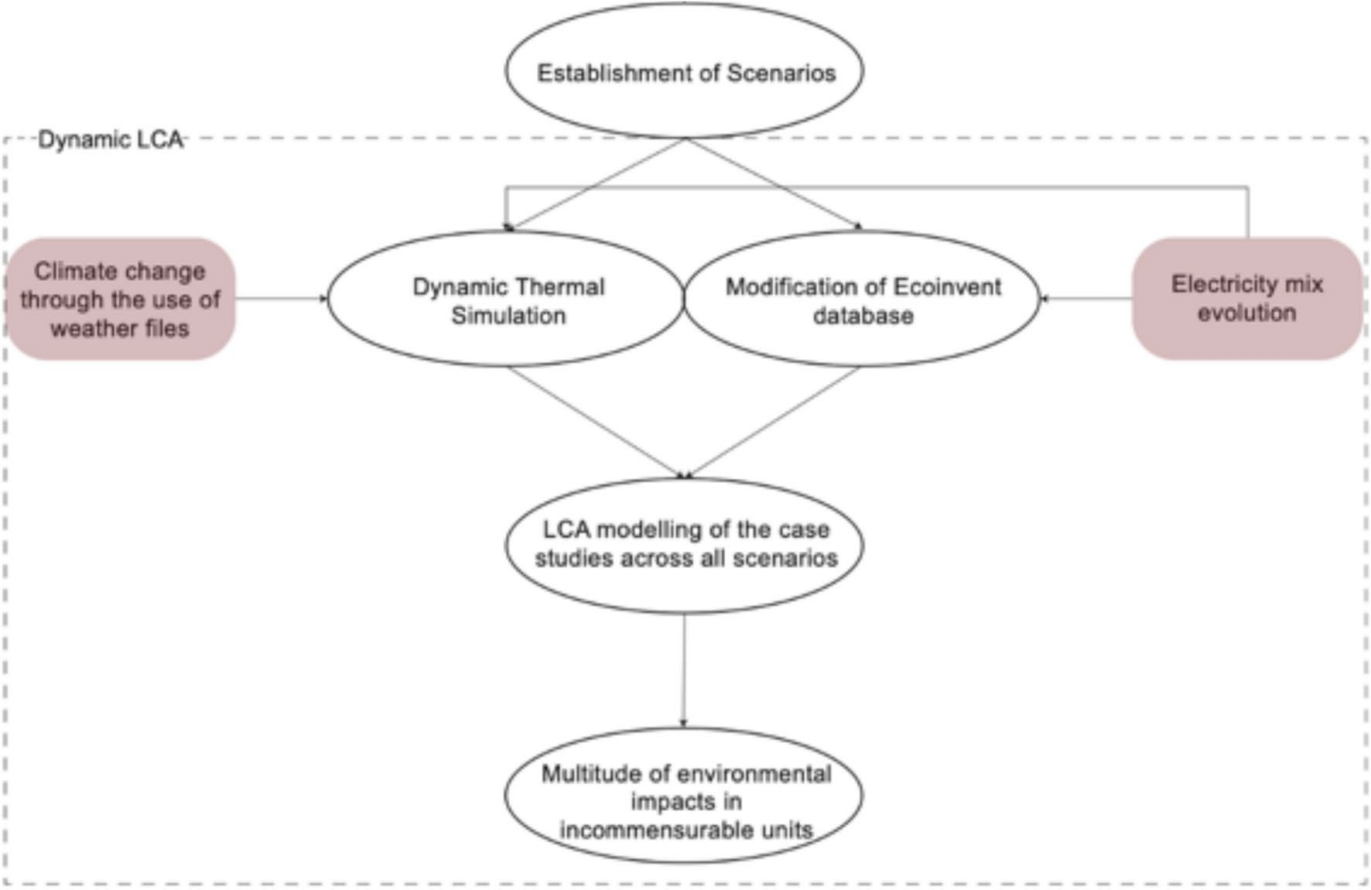
Methodology overview

Figure 2 illustrates the overall study flow that was followed.

**Fig. 3:**
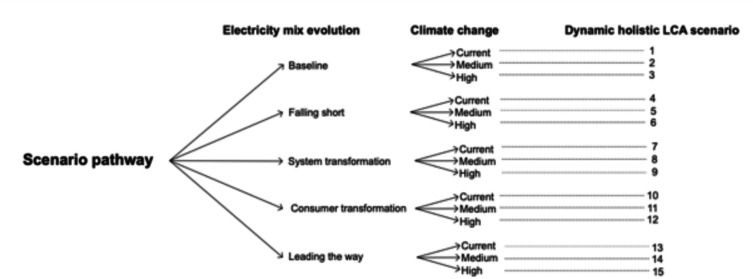
Simulated scenarios for the dynamic LCA

The first step of the study design was to establish the scenarios reflecting possible pathways for electricity mix and climate change; overall, 15 scenarios were constructed (Fig. 3). Scenario 13 represents the best case as it assumes no progression in climate change and attainment of a Net Zero electricity mix. Scenario 6 represents the worst case as little progress is made in electricity decarbonisation whilst climate change follows the worst trajectory.

Following the scenarios definition, modelling was carried on SimaPro 8.0.3 (PRé Sustainability 2014) and IESve (IES 2023). Since typical LCA tools do not allow modification in their Life Cycle Inventory (LCI), SimaPro 8.0.3 (PRé Sustainability 2014) was used to modify the Life Cycle Inventory (LCI) (Ecoinvent 3.01 (Ecoinvent 2013)) to reflect the electricity mix evolution pathways in the UK, the EU and China (for a detailed description, see Sect. 2.2). The dynamic thermal simulation of the case study building had been carried out on IESve (IES 2023) based on weather files reflecting London’s future trajectories for climate change (for a detailed description, see Sect. 2.3) with the intent to quantify the building’s operational energy demand at an annual level. After obtaining the operational needs and the material quantities for the case study, the LCA analysis has been carried out based on a modified Ecoinvent 3.01 (2013) inventory through the characterisation factors of ReCiPe (Huijbregts et al. 2016). This means that the LCA protocol has not been challenged in any way except for the consideration of climate change and varying electricity mixes.

**Fig. 4:**
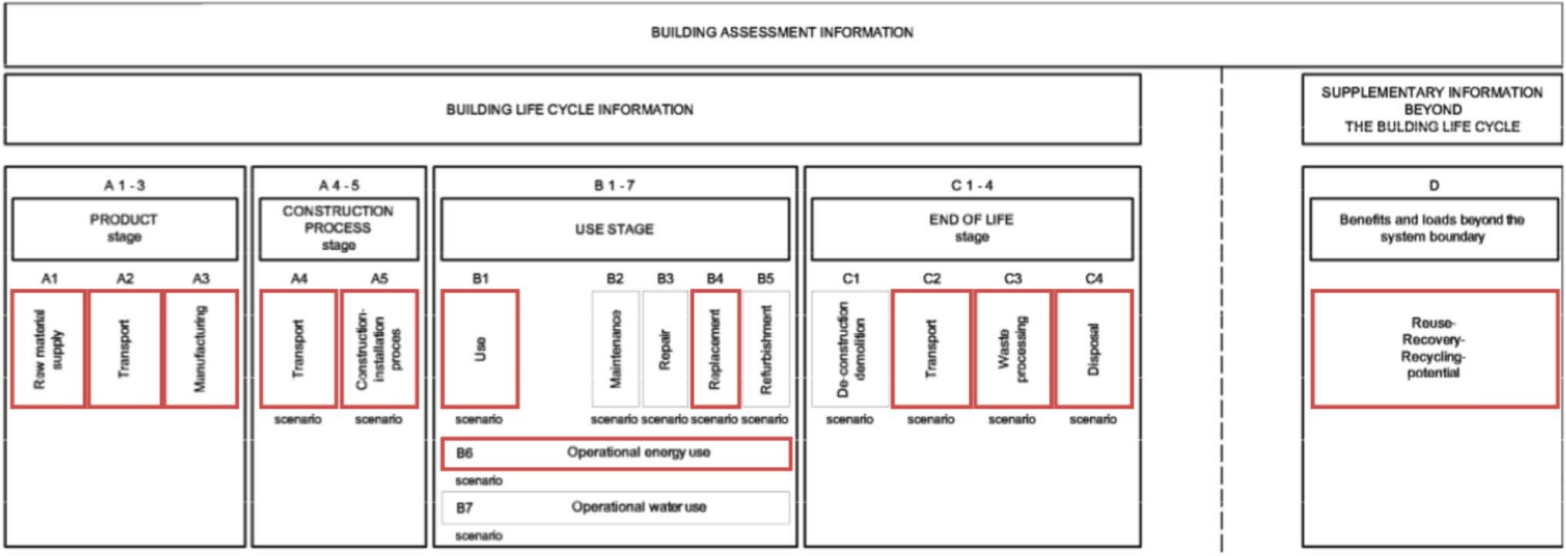
LCA modules calculated in this study (base sketch by BS EN (2021b))

Τhe evaluated LCA modules highlighted in Fig. 4 align the study’s system boundary with EN ISO 15978:2011 + A2:2019. Module D reflects the potential circular benefits of recycling materials at EoL. Due to its speculative nature, the results are presented separately from modules A to C (Mayor of London 2020; BS EN 2021b).

### Electricity mix evolution in LCA for embodied and operational modules

To explore the influence of electricity mix evolution on embodied (all highlighted modules except B6 in Fig. 4) and operational (module B6 in Fig. 4) environmental impacts of LCA, forecasted scenarios for the electricity mix had to be considered. For materials manufactured in the UK, the pathways developed by National Grid ESO (2022) were used: Falling Short (FS), Consumer Transformation (CT), System Transformation (ST) and Leading the Way (LW). For materials manufactured outside the UK yet within the EU, the current and future average EU electricity mixes derive from European Commission (2020a); for China, current and future electricity mix scenarios derive from Energy Foundation China (2015).

**Fig. 5:**
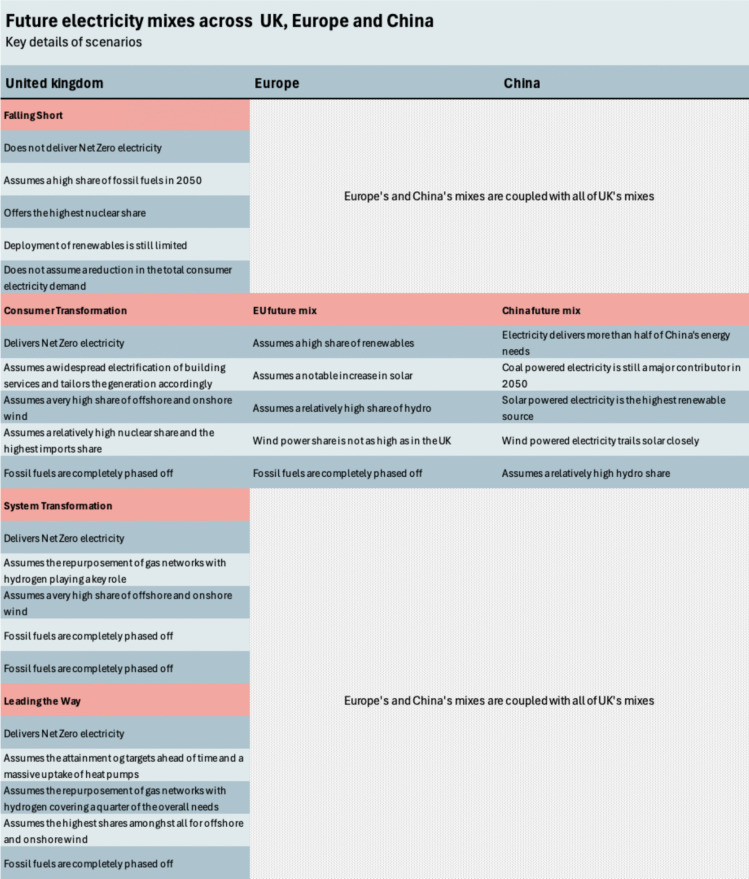
Coupling of regions’ electricity mixes

This study utilises different pathways for the electricity mix; therefore, future projections had to be matched (Fig. 5). As in the case of the EU and Chinese electricity mixes, no distinct scenarios are provided (Energy Foundation China 2015; European Commission 2020a); all UK future scenarios are matched with the forecasted trajectory of the European Commission (2020b) for the EU and the Energy Foundation China (2015) for China.

During simulation, the Ecoinvent 3.01 (2013) high, medium and low voltage databases in SimaPro 8.0.3 (PRé Sustainability 2014) were modified to capture current (as of 2024) and future average annual electricity mixes. For the UK, hydrogen-fuelled electricity and negative emissions from Bioenergy with Carbon Capture and Storage (BECCS) have not been considered as hydrogen has a minimal contribution (less than 1.5%), and BECCS is not yet on track for deployment (IEA 2024). To account for net imports from the EU when considering the UK consumption, Itten et al. (2012) had been followed. Net exports were ignored as per Itten et al. (2012) as they do not constitute part of the consumption. Therefore, imports have been considered for the consumption, whilst exports were ignored as they are not part of the consumption mix. The authors have considered using marginal mixes when conceptualising the methodology. However, unfortunately, there are no future projections available for the UK. Therefore, the more traditional annual average mixes have been used. As the case study (Sect. 2.5.1) implements in situ photovoltaics, the electricity consumption for heating, cooling and DHW accounts for the negative contribution of electricity generated by in situ photovoltaics. This has been done on IESve (IES 2023) when obtaining the results. Any surplus electricity exported to the grid was assumed to contribute to module D impact categories. This means that the amount of surplus electricity was multiplied with the grid’s annual impacts on a per kWh basis, leading to avoided impacts.

### Climate change in LCA through weather files for the operational module B6

To explore how sensitive the environmental impacts of module B6 are, CIBSE hourly weather files based on future projections of greenhouse gases (i.e. the UKCIP09 projections) were implemented (CIBSE 2021a) on IESve (IES 2023). CIBSE provides weather data relevant for 30-year periods (i.e. 2020 s, 2050 s and 2080 s). This study used the 90th percentile Test Reference Years (TRY) files as they give an indication of the extent of likely future warming with the probability of temperatures greater than those of the 90 th percentile being unlikely according to UKCIP09 (Eames et al. 2011). RCP 2.6 was recently evaluated as unlike considering the demographical projections and the magnitude of immediate energy system changes it requires (Jones and Warner 2016). Therefore, CIBSE medium and high emissions weather files were implemented on IESve to obtain the annual electricity consumption associated with all building services. As CIBSE (2021a) does not give medium emission weather files for the 2020–2050 period, this study averaged the 2050s medium and current weather results to obtain an indication of the potential energy consumption for 2020s medium climate. Internal heat gains, occupancy profiles, temperature setpoints and water consumption had been set in line with CIBSE Guide A (CIBSE 2015), ASHRAE Handbook of Fundamentals (ASHRAE 2021) and UK NCM (BRE 2022). Windows are not openable and therefore no natural ventilation was considered.

**Fig. 6:**
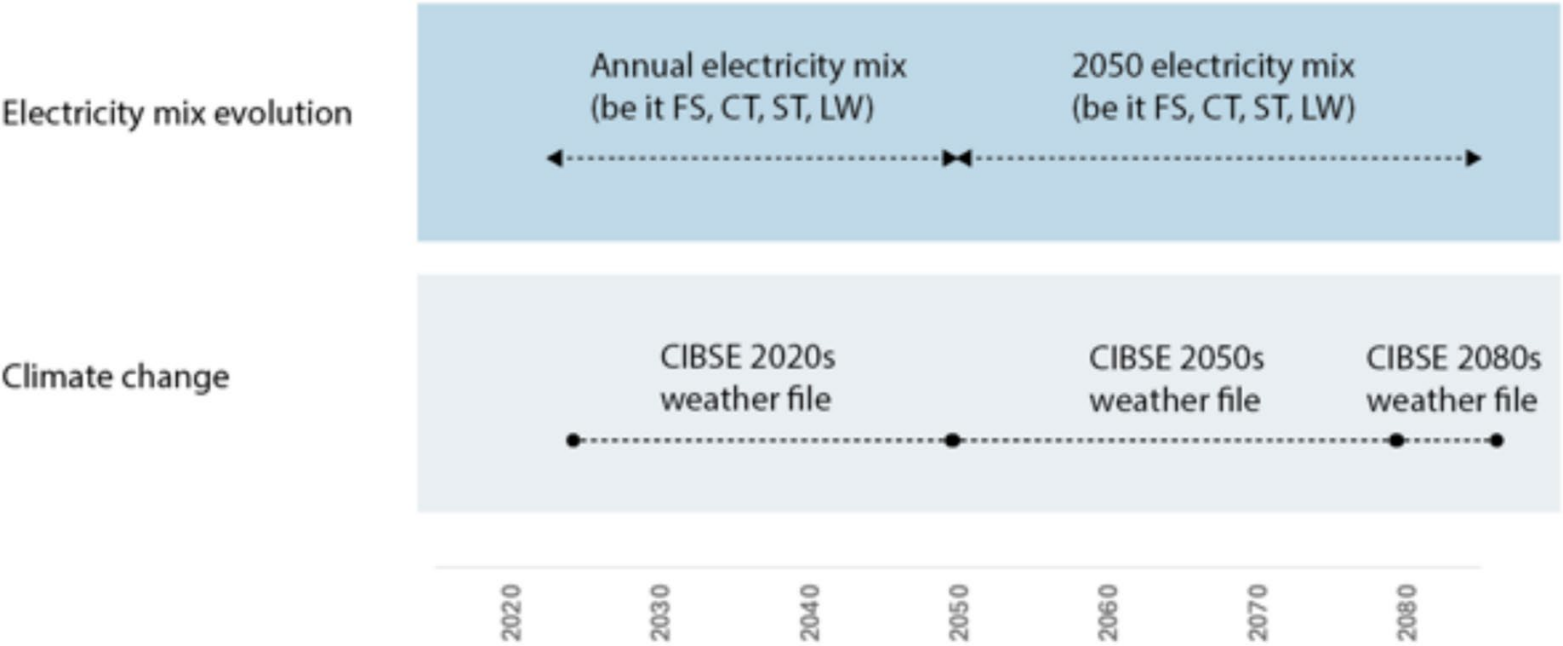
Illustration of the timeframe of the operational energy modelling considering climate change

Finally, the annual electricity consumption was translated into environmental impacts based on the modified Ecoinvent database on SimaPro. The annual electricity needs from 2024 to 2050 were translated into impacts by using the average annual electricity mix of the respective electricity scenario. Beyond 2050, needs made use of the 2050 mixes, assuming they would prevail for beyond 2050 years (Fig. 6).

### Environmental impact categories

In this study, the analysed environmental impacts (Table 2) were calculated in accord with the ReCiPe Midpoint (H) method (Huijbregts et al. 2016) developed on behalf of the Dutch Ministry of Infrastructure and the Environment. ReCiPe is considered a cutting-edge and up-to-date LCA methodology for studies in Europe, as it shares the same methods on most impacts with EN ISO documents (Çolak et al. 2022).

**Table 2:** Environmental impacts examined (Huijbregts et al. 2016)

Impact category	Unit	Brief description
Climate change (CC)	kg CO_2e_	Quantifies the emissions of GHG leading to increased radiative forcing capacity and global mean temperatures
Ozone depletion potential (ODP)	kg CFC_11e_	Quantifies the anthropogenic emissions of ozone depleting substances which decrease the atmospheric ozone resulting in increased UVB radiation
Terrestrial acidification (TA)	kg SO_2e_	Quantifies the atmospheric deposition of inorganic substances that cause a change in the acidity of soil
Freshwater eutrophication (FEU)	kg P_e_	Quantifies the discharge of phosphorus into soil or water bodies causing a rise in eutrophying nutrient levels
Marine eutrophication (MEU)	kg N_e_	Quantifies the runoff and leach of nitrogen into riverine and marine bodies causing a rise in eutrophying nutrient levels
Human toxicity (HT)	kg 1,4-DB_e_	Quantifies the emissions of toxic substances to urban air
Photochemical ozone formation (POF)	kg NMVOC	Quantifies the emissions of non-methane volatile organic compounds (NMVOC) which react with NO_x_ forming ozone
Particulate matter formation (PMF)	kg PM_10e_	Quantifies the emitted mixture in the air of organic and inorganic substances with a diameter of less than 10 µm that cause human health issues
Terrestrial ecotoxicity (TE)	kg 1,4-DB_e_	Quantifies the emissions of toxic substances to industrial soil
Freshwater ecotoxicity (FE)	kg 1,4-DB_e_	Quantifies the emissions of toxic substances to freshwater
Marine ecotoxicity (ME)	kg 1,4-DB_e_	Quantifies the emissions of toxic substances to seawater
Ionising radiation (IR)	kBq U235_e_	Quantifies the anthropogenic emissions of radionuclide
Agricultural land occupation (ALO)	m^2^/year	Quantifies the agricultural area occupied and the time of occupation in years
Urban land occupation (ULO)	m^2^/year	Quantifies the urban area occupied and the time of occupation in years
Natural land transformation (NLT)	m^2^/year	Quantifies the natural area transformed and the time of transformation in years
Water depletion (WD)	m^3^	Quantifies the total amount of water used from lakes, rivers, wells and unspecified origns
Metal depletion (MD)	kg Fe_e_	Quantifies the minerals extracted from a deposit
Fossil depletion (FD)	kg oil_e_	Quantifies the use of resources that contain hydrocarbons

### Study assumptions

#### Description of the case study

**Fig. 7:**
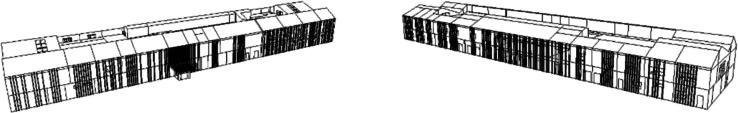
Sketch of the case study building

The proposed approach was tested on a 4044 m^2^ steel-framed multi-purposed office building (Fig. 7) with gym facilities in London, UK. Its material and design characteristics (concrete foundations, aluminium framed windows, polycarbonate roof and in situ photovoltaics, shading) ascertained that all research questions can be addressed.

Steel products and their impacts are highly influenced by the background electricity mix, especially if produced via the Electric Arc Furnace (EAF) route. Considering that EAF is the preferred manufacturing route for sustainable steel production due to the high recycled rates and lower carbon emissions (Hasanbeigi et al. 2016; European Commission 2022), it is worth investigating the influence of grid decarbonisation on steel’s embodied impacts. Similarly, studies indicate that 70–80% of the overall aluminium products’ emissions are due to the grid’s carbon intensity (Liu et al. 2011; Cullen and Allwood 2013; Daehn et al. 2021), and hence, a case study with aluminium framed windows would be ideal due to the many replacement cycles.

Concrete is at the epicentre of attention when it comes to low carbon buildings. The evolution of the electricity mix to ‘greener’ technologies is not largely translated into lower concrete embodied carbon, as half of concrete’s emissions are due to clinker’s chemical reaction that emits CO_2_ (Allwood et al. 2022). In response, research has suggested the use of high content of ground granulated blast-furnace slag (GGBS) in the concrete mix, which reduces the need for cement that can lead to lower embodied carbon emissions. However, the beyond carbon impacts of GGBS are not widely analysed, and it is essential to investigate whether the optimisation of carbon is achieved at the expense of other impacts. Finally, in situ photovoltaics, due to previously raised concerns around ecotoxicity impacts (UNECE 2021) and due to the replacement cycles of the mounting system, enable the investigation of beyond carbon impacts.

Overall, this case study represents a typical non-residential UK building that has taken steps towards lowering carbon emissions through material selection and shading yet is still short of carbon neutrality. Therefore, the findings drawn can be relevant to policy.

#### Building reference study periods and materials lifespan

The building lifespan is assumed to be 60 years as per RICS (2023). Therefore, building elements with a lifespan of less than 60 years were assumed to be replaced based on their expected lifespan (Table 3). The background electricity involved in processes of building materials (e.g. manufacturing and EoL) corresponds to the respective year the process takes place. Hence, all materials manufactured and installed today use the 2024 electricity mix, whereas the ones replaced in future use the mix (be it FS, CT, ST and LW) of the respective point in time. The system boundary of the case study covers the highlighted LCA modules (Fig. 4); module A5 reflects the excavation works alongside the construction waste for each element that has been calculated based on waste rates provided by RICS (2023).

**Table 3:** Expected lifespan of building materials (RICS 2017)

Building part	Building element	Lifespan
Roof	Roof coverings	30 years
Internal partitioning	Internal partitioning	30 years
Finishes	Floor finishes	30 years
	Raised access floor	10 years
	Ceiling finishes	10 years
	Wall finishes	10 years
MEP	Space heating systems	20 years
	Space cooling systems	15 years
	Ductwork	20 years
	Electrical installations	30 years
	Water installations	25 years
Façade	Opaque cladding (rain screens)	30 years
	Windows and external doors	30 years
	Curtain walling	35 years

All remaining materials not mentioned above have an assumed lifespan as per the building’s one

#### Transport

Transportation of products between the manufacturing and construction site is set at tkm, with required distances taken from RICS (2017). As tkm represents the transport of 1 tonne over 1 km (Eurostat 2023), each material’s quantity was multiplied by the respective distance of RICS. For scenarios utilising non-baseline electricity (Fig. 3), the diesel-fuelled trucks were assumed to be electrified using a consumption of 0.926 kWh per km (CENEX 2021). No changes in fuel were assumed for sea transport. For locally and nationally manufactured elements, the electricity used is sourced from the UK grid (be it FS, CT, ST, LtW), whereas for European manufactured ones, electricity was assumed to be EU average (Table 4).

**Table 4:** Transportation scenarios and distances (RICS 2017)

Transport scenario and materials	km by road	km by sea
Locally manufactured (e.g. concrete, gravel, sand and reinforcing steel)	50	-
Nationally manufactured (e.g. concrete block, ceramics, insulation, plasterboard, timber, glass and plastics)	300	-
European manufactured (e.g. cross laminated timber, façade modules, windows, carpet, heating and ventilation system and paint)	1500	-
Globally manufactured (e.g. specialist stone cladding, PVs)	200	10,000

For the EoL (module C2), the distance between the building site and the disposal unit was set as 50 km by road as per Norouzi et al. (2022). To avoid double counting, the transportation of materials that are to be recycled is considered in modules C as per BS EN (2021b).

#### End-of-life scenarios

By reviewing the UK and EU recycling rates of building materials (European Commission 2018) and considering the EoL scenarios by RICS (2023) and Norouzi et al. (2022) for UK landfill, recycling and incineration, the proportions presented in Table 5 were hypothesised. Landfill and incineration contribute to module C, whilst the benefits of recycling are accounted for under module D. This means that no recycling rate was already accounted for in module A of materials. The results of module D were reported separately in line with EN15978.

**Table 5:** End-of-life scenarios for building materials (European Commission 2018; Norouzi et al. 2022; RICS 2023)

Materials	End-of-life rates		
	Landfill	Recycling	Incineration
Plywood, wood chipboard, hardboard, softwood	25%	0%	75%
Reinforcing steel, stainless steel, steel, galvanised steel	4%	96%	0%
Copper, brass	35%	65%	0%
Aluminium	4%	96%	0%
Glass	75%	25%	0%
Electronics, wiring	50%	50%	0%
Plastics	0%	0%	100%
Cement mortar, plaster coat, ceramic tiles, brick, concrete block, plaster boards	10%	90%	0%
Polystyrene, polyurethane, XPS, polyethylene			
PE, PP, LDPE pipes, PS			
PVC			

For MEP equipment, the EoL was based on their constituent materials and the rates described in this table. The inventories were sourced from Naumann et al. (2022) for heat pumps, BSRIA (2014) for indoor FCUs and IEA (2020) for photovoltaics

#### Building fabric and material thermal properties

All material thermal properties use actual case study data except for the doors and internal partitions that are set as per the UK building regulations (Part L) (The Building Regulations 2021) (Table 6).. The infiltration rate was set at 5 ${m}^{3}/h.{m}^{2}$ as per Part L (The Building Regulations 2021) which is equivalent to 0.125 ACH as per CIBSE TM23 (CIBSE 2022). Internal heat gains and occupancy schedules are outlined in Table 9 of the Appendix, following ASHRAE (2021) and UK NCM (BRE 2022). Temperature setpoints span from 21 to 23 °C for all rooms (Table 9 of Appendix), except for the gym and corridors (18 °C).

**Table 6:** Building fabric and *U*-value of elements

Element	Layer	Thickness (mm)	Thermal conductivity (${~}^{{\boldsymbol{W}}}\!\left/ \!{~}_{{\boldsymbol{m}}.{\boldsymbol{K}}}\right.$)	*U*-value (${~}^{{\boldsymbol{W}}}\!\left/ \!{~}_{{{\boldsymbol{m}}}^{2}.{\boldsymbol{K}}}\right.$)
Rooflight	Outer pane	8	0.03	1.6
	Cavity	12	-	
	Inner pane	8	0.03	
Roof	Bitumen capping sheet	5.2	0.23	0.14
	Bitumen underlayer sheet	3	0.23	
	Polyisocyanurate insulation	154.9	0.022	
	Bitumen vapour control	2.5	0.23	
	Reinforced concrete	150	2.5	
Internal partitions	CLT panels layer 1	50	0.21	0.24
	Rockwool insulation	150	0.035	
	CLT panels layer 2	50	0.21	
Ground and internal floors	Bitumen membrane	0.9	0.1	0.2
	Polyisocyanurate insulation	30	0.0065	
	Polyethylene separation layer	0.5	0.01	
	Reinforced concrete	175	2.5	
	Screed	70	1.4	
External window	Outer aluminium pane	6	0.032	1.45
	Cavity	16	-	
	Inner aluminium pane	6	0.032	
External wall	Gypsum plasterboard	30	0.19	0.19
	Airspace	15	-	
	Reinforced concrete	140	1.13	
	Rockwool insulation	150	0.034	
	Ventilated cavity	50	-	
	Polycarbonate cladding	-	-	
Doors	-	-	-	1.6

[1] Windows and rooflights *U*-value also account for the frame consisting of a *U*-value of 2 W/m^2^.K

[2] Heat pumps COP efficiency was set at 3.5

[3] Heating efficiency of FCU was set at 4.5

#### Material quantities

The material quantities are presented in Table 7. Heat pumps were modelled using the inventory of Naumann et al. (2022) assuming a 6% R134a refrigerant leakage per year to be replaced annually on top of the upfront 4.9 kg. As their inventory reflects heat pumps with a heating capacity of 5 kW, the inventory had to be modified to model the 280 kW of the case study on a per functional unit basis of 5 kW. Material data for indoor units derive from BSRIA (2014). The inventory of photovoltaics has been sourced from IEA (2020) assuming a total 12.51 kg/m^2^ with 11 kg of unframed weight and 1.51 kg of frame (Müller et al. 2021). The mounting system for the PVs was modelled in accord with IEA (2020); 5.04 kg of aluminium and 5.01 × 10^−1^ kg of EAF steel for those placed on a flat roof, and 5.68 kg of aluminium and 3 kg of EAF steel for those placed on a slanted roof (IEA 2020). All materials were assumed to be sourced from the UK, except for heat pumps, indoor units and inverters that were sourced from the EU according to BEIS (2020). PVs were sourced from China, as per IEA (2022), and their mounting system from the UK.

**Table 7:** Material quantities used in the LCA modelling

Element typology	Material	Quantity
Substructure	Excavation	263.68 m^3^
	Reinforcement bars, EAF route, hot rolled	50 tonnes
	Polyisocyanurate insulation	10.3 tonnes
	Polyethylene separation layer	0.3 tonnes
	Sand	313.5 tonnes
	Cement screed	90.7 tonnes
	Concrete with 50% GGBS content	1,959 tonnes
Superstructure	Gypsum plasterboard	14.7 tonnes
	Polyisocyanurate insulation	6.17 tonnes
	Polycarbonate roof and walls cover	57.4 tonnes
	Rockwool insulation	14.8 tonnes
	Bitumen membrane sheets	3.5 tonnes
	Roof tiles	15.1 tonnes
	Precast concrete	5 tonnes
	Concrete with 50% GGBS content	837 tonnes
	BOF steel	345.6 tonnes
	EAF steel	8 tonnes
	Softwood	21.6 tonnes
	Glulam	4 tonnes
	CLT timber	45.5 tonnes
	Polyurethane insulation	0.5 tonnes
	Double glazed aluminium framed windows	226.5 m^2^
MEP	Air source heat pump	280 kW
	R134a refrigerant	4.9 kg
	Indoor fan coil units	37 units
	Single silicon photovoltaics	992 m^2^
	Inverter	48 kW
	MVHR	4 units
	Aluminium mounting system for PVs	5.2 tonnes
	EAF steel for PVs mounting system	tonnes

## Results and discussion

This section presents and discusses the results of lifecycle environmental impacts of dynamic LCA relative to the baseline LCA, which represents a static approach both for climate and electricity mix (scenario 1).

### Energy consumption

**Fig. 8:**
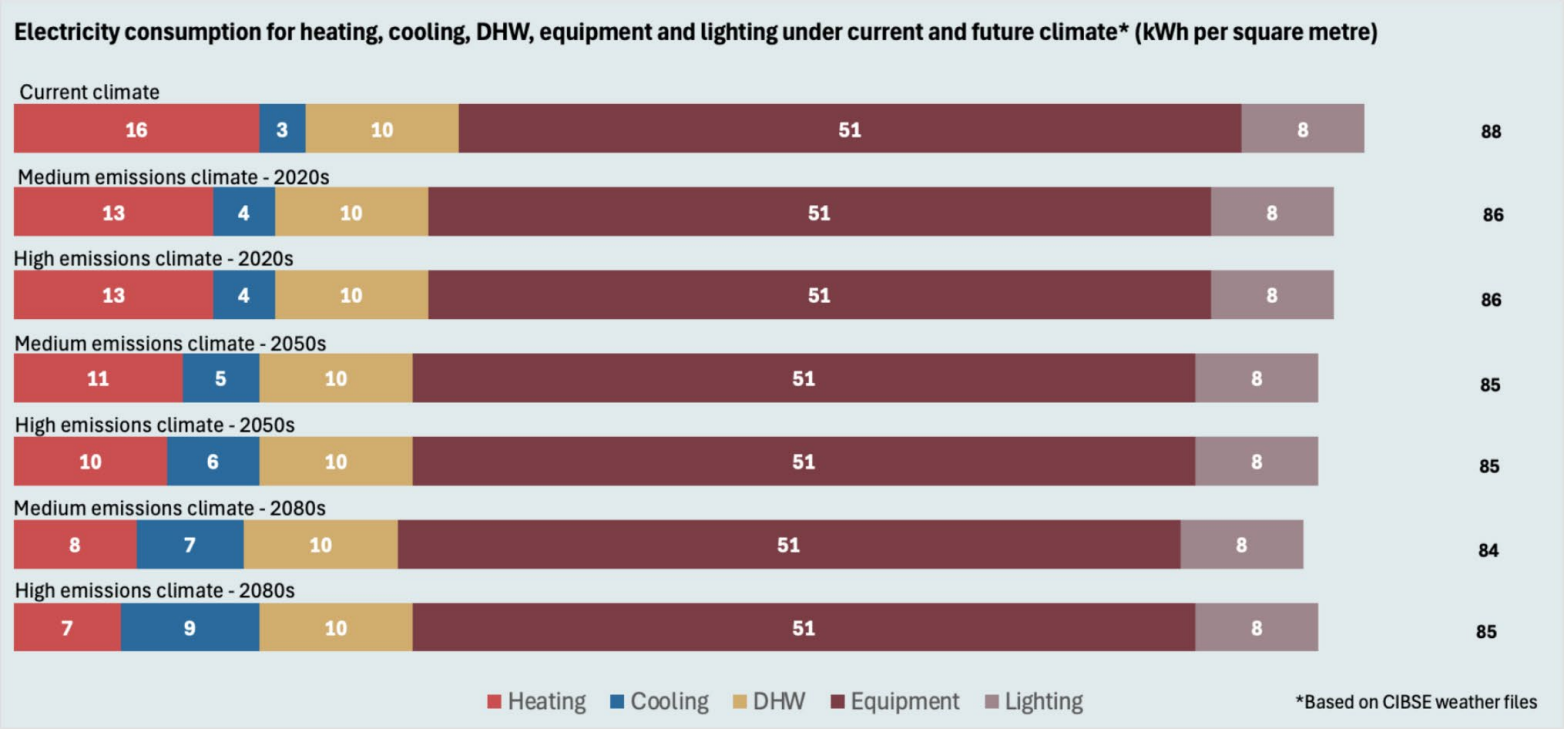
Electricity consumption of module B6 for current and future climate

The operational electricity needs are presented in Fig. 8. Cooling was considered only for summer months (May–September). Using climate change data (CIBSE medium and high climate) leads to reductions in heating demand, which accords with previous studies (Roux et al. 2016; Morshed and Mourshed 2023). Despite implementing solar protection in the case study, cooling loads increased due to warming temperatures. Overall, there are minor reductions in electricity consumption, indicating that no significant savings are to be expected simply because of climate change if the building is actively cooled. The relatively high share of DHW is due to hot water needs for the gym showers and laundry. The total consumption is below CIBSE’s good practice benchmarks for actively cooled offices (128 kWh per metre squared) and changing grounds facilities (93 kWh per metre squared) (CIBSE 2021b).

### Results for the lifecycle environmental impacts

Table 8 presents the results of the baseline scenario, which represents a static LCA (i.e. without electricity evolution or climate change considerations).

**Table 8:** Environmental impacts for the static LCA baseline scenario over 60 years

Impact category	Abbreviation	Units	Embodied (per m^2^)	Operational (per m^2^)	Module D (per m^2^)
Climate change	CC	kg CO2_e_	1167	954	− 263
Ozone depletion	OD	kg CFC-11_e_	0.0048	0.0002	0.0000
Terrestrial acidification	TA	kg SO2_e_	4.75	3	− 1
Freshwater eutrophication	FEU	kg P_e_	0.50	0.26	− 0.07
Marine eutrophication	MEU	kg N_e_	0.43	0.16	− 0.04
Human toxicity	HT	kg 1,4-DB_e_	869	505	− 62
Photochemical oxidant formation	POF	kg NMVOC	4.35	9	− 1
Particulate matter formation	PMF	kg PM10_e_	2.60	0.98	− 1
Terrestrial ecotoxicity	TE	kg 1,4-DB_e_	0.29	0.19	0.00
Freshwater ecotoxicity	FE	kg 1,4-DB_e_	25	23	− 1
Marine ecotoxicity	ME	kg 1,4-DB_e_	24	23	− 1
Ionising radiation	IR	kBq U235_e_	81	1001	− 5
Agricultural land occupation	ALO	m^2^a	70	51	− 2
Urban land occupation	ULO	m^2^a	14	17	− 2
Natural land transformation	NLT	m^2^a	0.11	0.40	− 0.02
Water depletion	WD	m^3^_e_	1804	1174	− 376
Metal depletion	MD	kg Fe_e_	622	155	− 113
Fossil depletion	FD	kg oil_e_	248	352	− 67

**Fig. 9:**
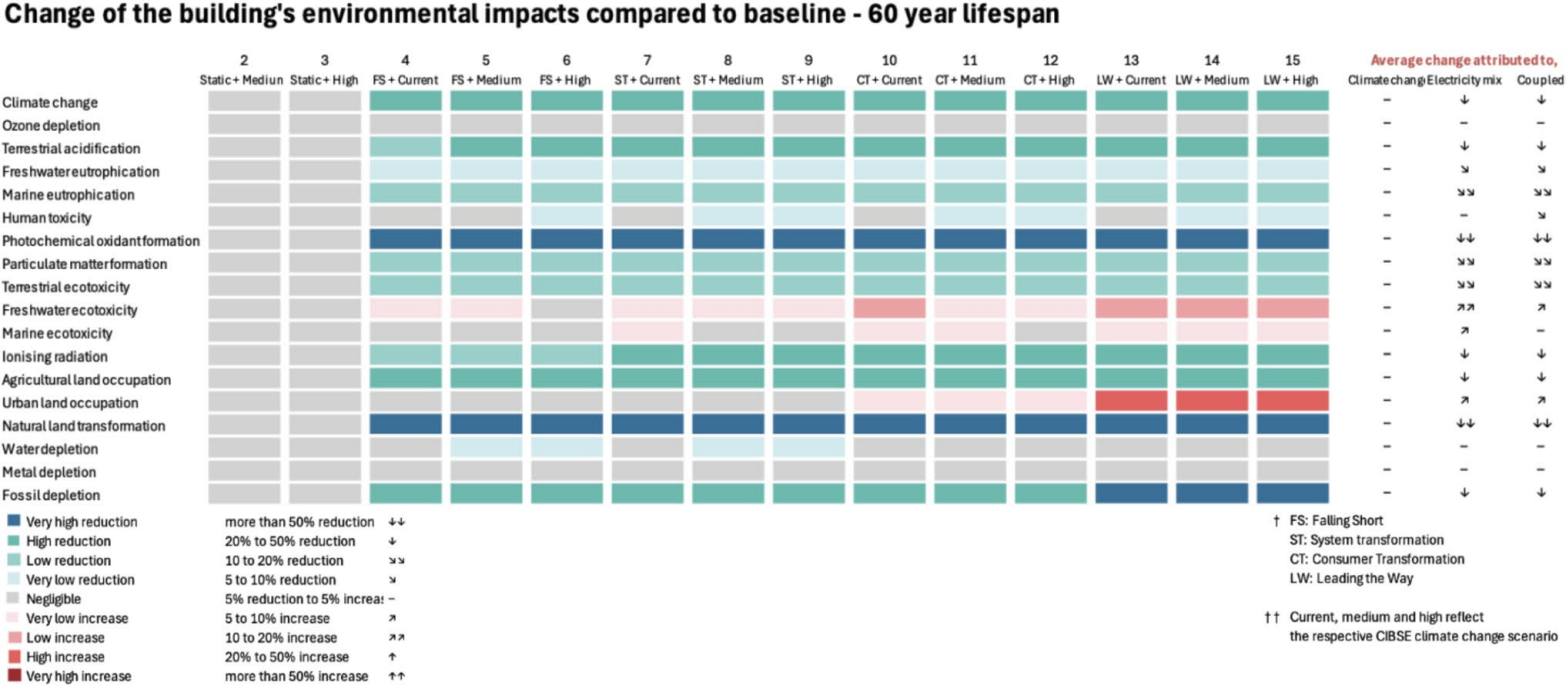
Lifecycle environmental impacts of the modelled scenarios against the baseline

Figure 9 presents the changes in the case building’s lifecycle impacts compared to baseline (Table 8) when considering the scenarios of Fig. 3. Differences span from very high reduction (more than 50% reduction) to negligible (+/− 5%) and very high increase (more than 50% increase). This presentation allows for distinguishing the effects of climate change and grid decarbonisation on the case’s LCA. It is worth reminding that the aim of the study, as expressed in Sect. 1.2, is to explore how evolutions in the electricity mix and climate change affect the LCA results of buildings across the multitude of environmental impacts. A comparison of different electricity production scenarios is not the aim of the study per se, as this would go beyond building LCA because electricity production influences other sectors (transport, industry, etc.) and because LCA does not account for risks (e.g. related to nuclear plants) and long-term impacts (e.g. related to radioactive waste).

There are several co-benefits of grid decarbonisation when it comes to the environmental impacts of the case study. However, freshwater, marine ecotoxicity and urban land occupation come to light as impacts with forecasted trade-offs. OD and MD are not influenced by grid decarbonisation as they remain unchanged across all scenarios. The general direction towards mitigating climate change whilst achieving a fast-paced decarbonisation offers a better environmental performance of the case building across most impacts.

Since climate change did not practically change the electricity needs (Fig. 8), results are therefore more sensitive to the electricity mix. Most embodied impacts in dynamic LCA reduce by 5%, except for carbon and TA that reduce by almost 10%, and IR that increases by almost 10% due to the electrification of transport (Appendix). This indicates that when the replacement phase has a relatively low contribution to embodied impacts, reduction of lifecycle impacts cannot rely on future grid decarbonisation and electrification of transportation. Hence, focus on minimising upfront impacts through early design choices is important. The electrification of transport is forecasted to increase embodied IR due to electricity’s higher IR impact compared to petrol-fuelled trucks. Therefore, sourcing materials locally to minimise transportation distances is important. Last but not least, the potential benefits from module D (Table 8) suggest that recycling is an important step towards a circular economy, and therefore, ensuring high recyclability rates at EoL is important.

### Etiology behind the trends of environmental impacts

Figure 9 shows that grid decarbonisation is the driver behind the observed changes, as had already been suggested by Fig. 8. Therefore, the magnitude of change for each impact category depends on the share of embodied and operational modules.

**Fig. 10:**
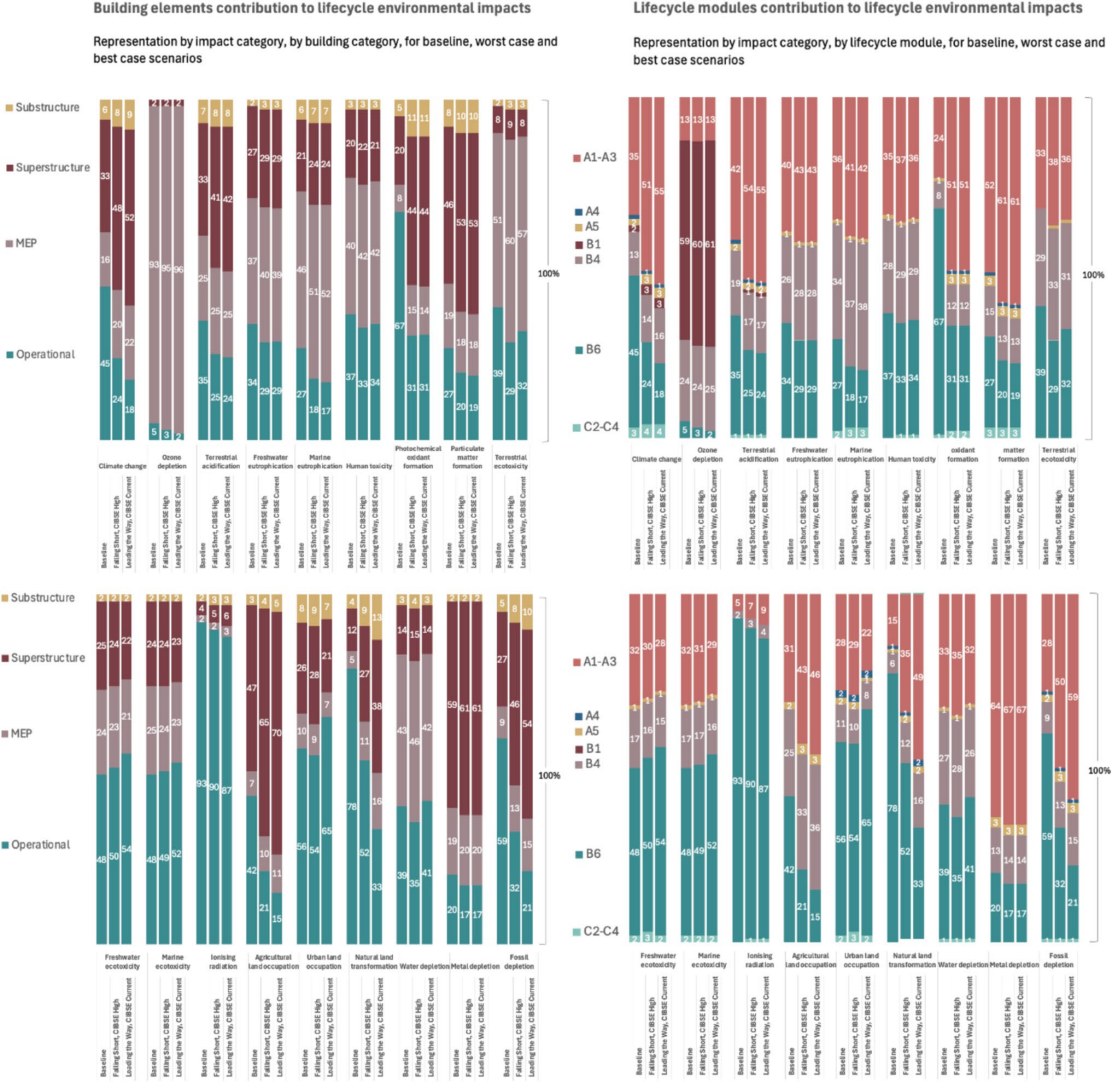
Lifecycle environmental impacts by building element and lifecycle module for the baseline, worst case and best case scenarios

**Fig. 11:**
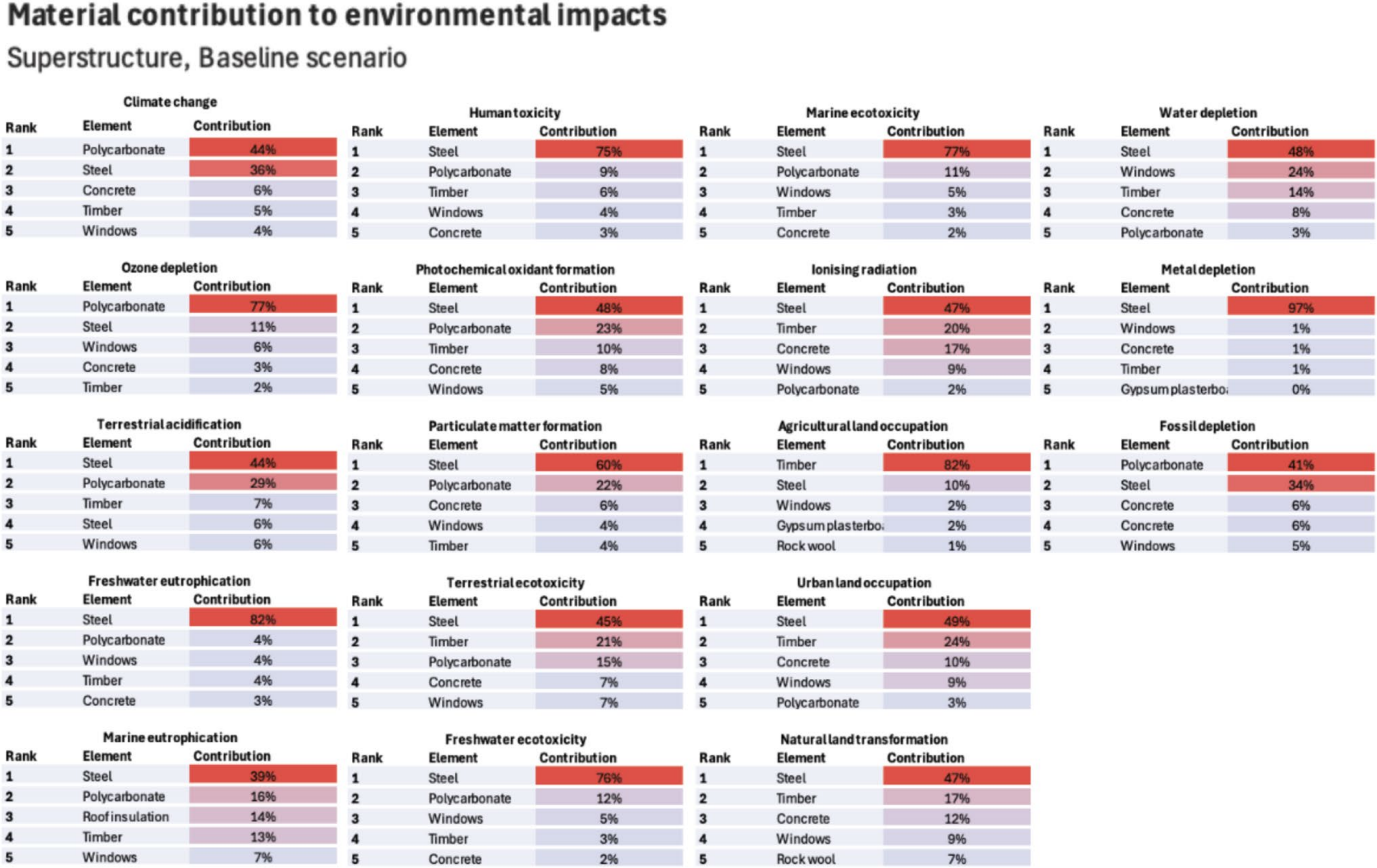
Superstructure material contribution to embodied environmental impacts

**Fig. 12:**
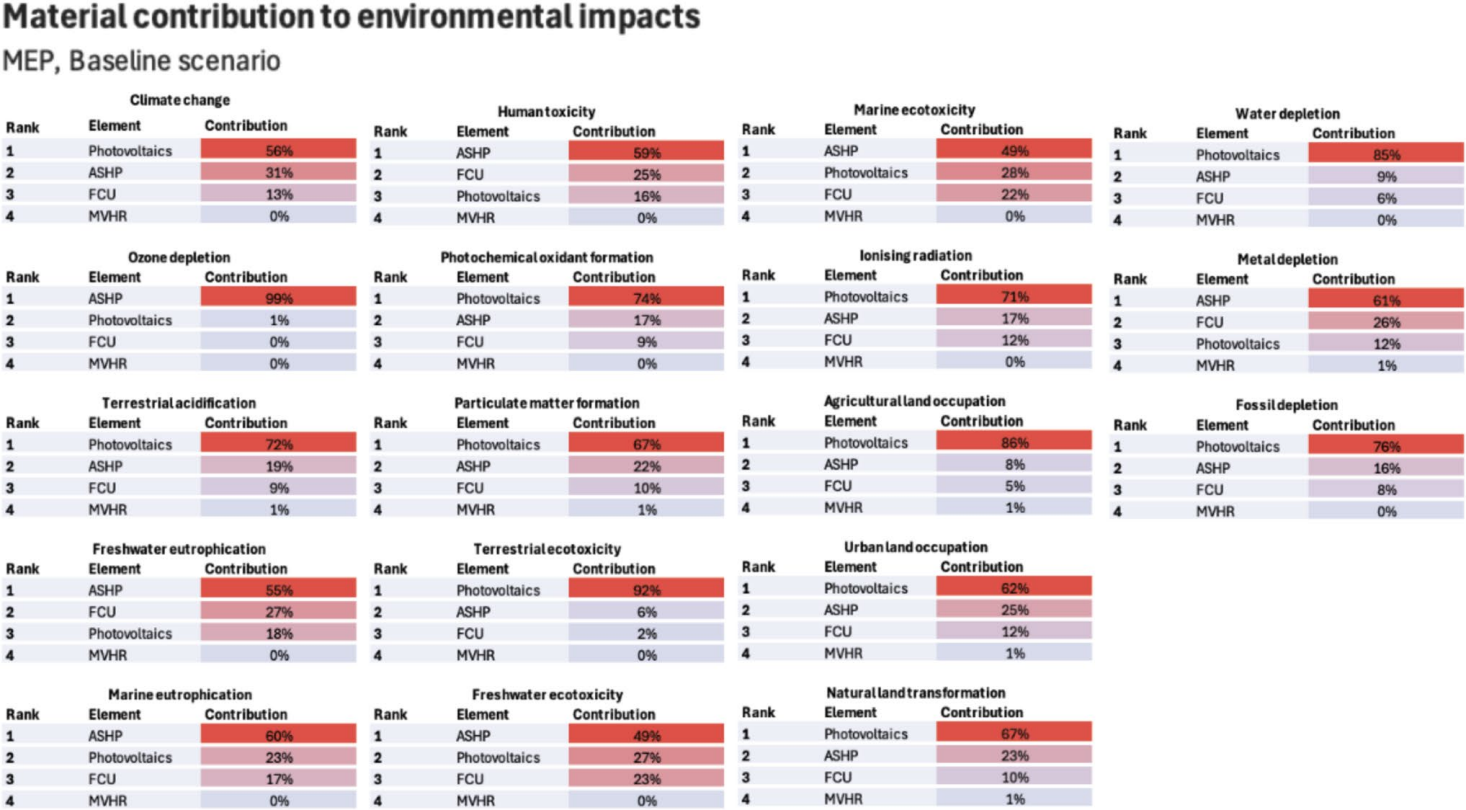
MEP material contribution to embodied environmental impacts

The share of operational and embodied LCA modules are illustrated in Fig. 10 for the baseline, the worst (scenario 6) and the best case (scenario 13) for RICS (2023) element typologies and LCA modules (Fig. 4). Overall, modules A1–A3, B4 and B6 stand out as the most contributing ones across all impacts, except for OD in which refrigerant refill leads to high B1 contribution. Modules A4, A5 and C2–C4 have very little influence across all impacts. The relative contribution of module B6 reduces in future across most impacts, except for FE, ME and ULO. This is because all building’s impacts benefit from grid decarbonisation, except for these three. Finally, Figs. 11 and 12 identify the most contributing materials and elements for each impact for superstructure and MEP. Due to the insignificant changes in embodied impacts, the shares of Figs. 11 and 12 did not change from electricity evolutions.

Sections 3.3.1 and 3.3.2 explore the etiology for the trends of environmental impacts of Fig. 9 by considering the contribution of building elements and LCA modules.

**Fig. 13:**
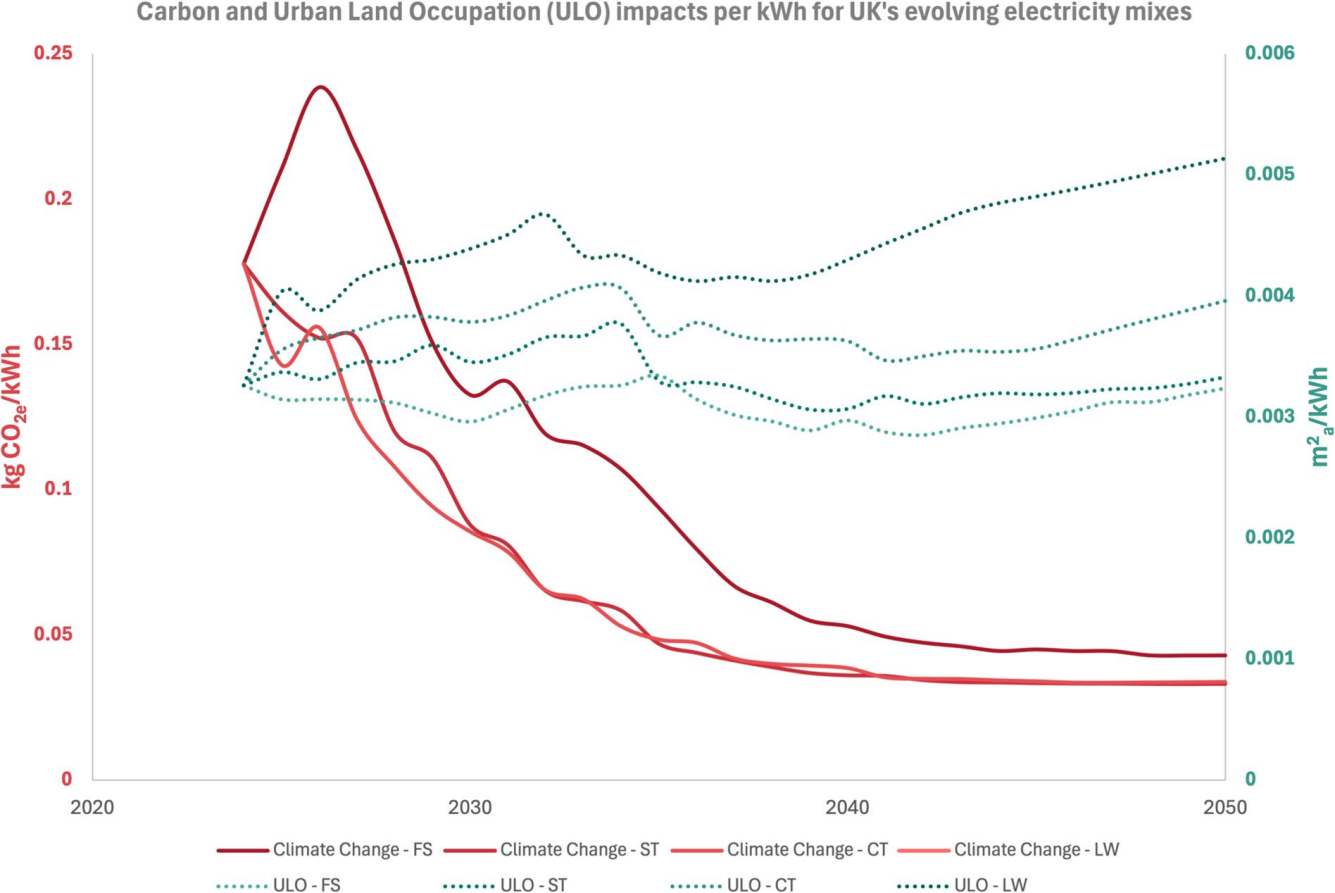
Carbon and ULO impacts per kWh for UK’s evolving electricity mixes

Finally, Fig. 13 helps associate two indicative environmental impacts (carbon and ULO) with each unit of electricity used, whilst the Supplementary Information provides the conversion factors for the rest of the environmental impacts for each electricity mix scenario.

#### Environmental impacts building designers can influence

When it comes to embodied impacts, agricultural land occupation (ALO) and ozone depletion (OD) stand out. ALO benefits from grid decarbonisation as the phase-out of biomass helps the case building reduce its ALO footprint through module B6. Timber was responsible for 80% of superstructure’s ALO footprint, highlighting the importance of sourcing timber from producers that are not expanding their forestry into marginal agricultural land or are involved in illegal logging in areas that are not reforested.

Ozone depletion (OD) remains unchanged in dynamic LCA. OD is caused by the R134a refrigerant of heat pumps (modules A1–A3) and the need for annual refill (module B4) which has been shown before (Naumann et al. 2022). Research suggests that R290 refrigerant not only has a lower carbon footprint compared to R134a but also negligible OD (Choudhari and Sapali 2017; Yadav et al. 2022). Therefore, R290 heat pumps should be chosen where possible. Superstructure’s OD footprint is due to polycarbonate materials used in the roof and walls. Although no literature exists for OD impacts of polycarbonate materials, since they are petroleum-based products (Zhou et al. 2023), it can be argued that their footprint stems from on-site emissions through catalytic cracking and refining stages (Liu et al. 2020).

The building’s photochemical oxidant formation (POF) benefits from grid decarbonisation. UK’s electricity mixes deflect off natural gas moving towards offshore and onshore wind and photovoltaics (Fig. 1) that have lower NO_x_ and CH_4_ footprint (Luderer et al. 2019; UNECE 2021), materialising in a lower module B6 POF. Upfront POF is due to polycarbonate materials, timber, concrete and steel (Fig. 11). Timber’s contribution is rooted in logging, reforesting, debarking, sawing and transportation, all of which are processes making use of fossil fuels (Sultana et al. 2022). Therefore, it is critical to source timber from manufacturers that engage in sustainable practices with the least possible use of fossil fuels throughout A1–A3. Steel and concrete have a moderate POF footprint (Jang et al. 2022), whilst polycarbonate has a high upfront POF due to material usage and injection moulding processes (Cheung et al. 2017).

Literature shows that lifecycle carbon of buildings is to reduce as electricity mixes depend more on renewables (Su et al. 2020; IEA 2023a). This is confirmed in the case building as module B6 sees reductions, regardless of the assumed decarbonisation pathway. Even though the names of electricity scenarios suggest otherwise, the carbon emission differences between Falling Short and Leading the Way are not that significant (around 15%) as shown in Fig. 13. Therefore, all scenarios making use of dynamic electricity show reductions within the band of 20–50% reductions. Embodied carbon slightly reduces by almost 10%. Polycarbonate materials, steel, concrete, timber and in situ photovoltaics contribute to it. The relatively low contribution of module B4 shows that carbon reductions cannot rely on future grid decarbonisation, and actions should focus on modules A1–A3.

ASHP and on-site PVs explain MEP’s impact on marine eutrophication (MEU) due to dross from the aluminium smelting and electronic components that, through wastewater treatment, end up in water bodies (ICA 2023). When it comes to superstructure, BOF steel is a major contributor, suggesting that EAF should be preferred. Freshwater eutrophication (FEU) results show a similar reducing trend as MEU, with steel, ASHP and FCU being the main contributors.

The building’s natural land transformation (NLT) sees significant reductions. The UK grid is to become less land transformative, and the high share of module B6 translates that into a lower building’s NLT. EAF steel should be preferred to avoid the use of pig iron, whilst timber should be sourced from producers with the right forest management practices. This is to ensure the A1–A3 NLT of BOF steel and timber is minimised.

Fossil depletion (FD) has a large share of module B6, meaning that the deflection off fossil fuels helps the case building reduce its impact. However, polycarbonate and steel materials contribute considerably to A1–A3 FD. Polycarbonate materials have a dominating FD impact as they are made of petroleum derivatives (Zhou et al. 2023). Steel’s contribution is explained by BOF’s route dependence on fossil fuels (Nurdiawati et al. 2023) and pig iron, highlighting that BOF steel should be avoided where possible.

The building’s terrestrial acidification (TA) benefits from grid decarbonisation through module B6, as technologies with acidifying emissions are phased off (Luderer et al. 2019). As for embodied modules, polycarbonate materials and photovoltaics are large A1–A3 and B4 contributors. Polycarbonate materials, being petroleum-based, are acidifying contributors due to catalytic reforming taking place in the refining process (Liu et al. 2020). Acidification impacts of photovoltaics lie in manufacturing and are largely due to the high share of coal-fuelled electricity assumed in this study for China. However, as the impacts of Chinese PVs depend on each province’s processes (An and Sun 2024), using average electricity mixes for the whole of China might not realistically capture the fabrication impacts of PVs.

Human toxicity (HT) slightly reduces in dynamic LCA with A1–A3 and B4 modules having a very high share. MEPs contribute through FCUs, as they entail human toxicity impacts even in decarbonised manufacturing processes (Peukes et al. 2023; Litardo et al. 2023). Silicon feedstock processes explain the HT of PVs (Lunardi et al. 2018).

Particulate matter formation (PMF) reduces due to grid decarbonisation, for reasons discussed in Rueda-Bayona et al. (2022). Polycarbonate materials are responsible for superstructure’s PMF impact due to direct air emissions from coal combustion and bisphenol production (Zhou et al. 2023).

Terrestrial ecotoxicity (TE) decreases through module B6 as the grid decarbonises. The embodied water depletion (WD) stems mainly from on-site PVs, as there are large water needs for the manufacturing of silicon cells (UNECE 2021). As it is unclear whether the building’s WD will benefit from grid decarbonisation, it is important to source PVs manufactured in geographical locations where water scarcity is not an issue. However, using electricity from in situ PVs can reduce the lifecycle WD, as some of the grid’s generation technologies, such as nuclear, have a higher water footprint per kWh compared to PVs (UNECE 2021). As for the WD of BOF steel, this is largely due to pig iron and the needs for water in its manufacturing. This highlights once again that selecting EAF steel where possible is necessary.

Metal depletion (MD) did not reduce in dynamic LCA, and some consider it amongst the trade-offs of decarbonisation (Sacchi et al. 2022; Šimaitis et al. 2025). Therefore, minimising A1–A3 MD impact through material selection is essential, which in this case building means avoiding BOF steel. EAF steel with the highest possible recycled content should be sought, as it avoids ferromanganese, ferronickel, ferrochromium metals and pig iron that are used in BOF routes. The magnitude of the obtained MD, however, is largely dependent on the method used to model recycling. In this study, recycling was purposively considered in module D (instead of A) which is assessed separately to highlight the importance of reducing upfront material consumption.

Freshwater ecotoxicity (FE) and marine ecotoxicity (ME) have seen increases due to grid decarbonisation through module B6. Offshore wind (more than 50%) generates electricity through turbines made of aluminium, steel, copper and iron, all of which are materials provoking ecotoxicity impacts (Rueda-Bayona et al. 2022). Embodied FE and ME are caused by photovoltaics, ASHP, FCUs, aluminium and BOF steel. FCUs ecotoxicity impacts are due to copper and electronic components because of the metal mining activities (ICA 2023). Silicon wafers are the main contributors to the FE and ME of photovoltaics due to dichlorobenzene emissions (Lunardi et al. 2018). The aluminium mounting system, due to its electricity intensive manufacturing, provokes ecotoxicity impacts as well. Therefore, long lifespans to avoid B4 impacts should be sought. BOF’s contribution lies in steel slag, ferronickel and pig iron, which accords with previous research (Foekema et al. 2021). This means that BOF steel should be avoided, as it contributes to impacts for which burden shifting has been observed. As with MD, the method used to model recycling influences the magnitude of FE and ME.

In sum, all building’s impacts benefit from grid decarbonisation through module B6 except for FE, ME and ULO. However, there are some learnings relevant to building designers. Long lifespans can help reduce the contribution of B4 impacts, especially for photovoltaics and their aluminium mounting system. Polycarbonates materials influence impacts in a similar way as fossil fuels due to being petroleum-refinery products, and therefore, their use should be reavaluated. Materials and processes in the inventory of BOF steel contribute to impacts with trade-offs, and therefore, EAF steel should be preferred. Heat pumps with R290 refrigerants are desired if possible, as research says they have lower OD and carbon impacts compared to R134a ones. Finally, timber should be sourced from producers that do not expand their practices in marginal agricultural or forest land.

#### Environmental impacts on which building designers have a limited influence

This section explores the etiology for the trends of impacts that building designers have a limited influence on: ionising radiation (IR) and urban land occupation (ULO).

IR reduces through module B6 as UK’s grid reduces its nuclear dependency. As nuclear energy is the only technology that uses radioactive material as its main fuel (Goedkoop et al. 2009; UNECE 2021), there is little space to influence IR.

ULO increases when the building sources electricity from mixes with relatively high solar electricity. As ULO quantifies the land occupation of all processes associated with the building’s lifespan (Goedkoop et al. 2009), the increases are through module B6 due to grid’s solar powered electricity. This is due to photovoltaics’ relatively high land use per kWh compared to natural gas (UNECE 2021) when it comes to electricity generation. The increasing trend of ULO in future electricity mixes is highlighted in Fig. 13. However, two actions can help mitigate this observed burden shifting; firstly, sourcing electricity from PVs installed on building rooftops instead of ground mounted ones. Secondly, the surplus of in situ generated electricity might be able to balance lifecycle ULO and should be sought if possible as exports to the grid can offset ULO under module D. Pig iron is responsible for BOF steel’s ULO footprint, which calls for the use of EAF steel to reduce A1–A3 ULO impacts.

### The emergence of a performance gap for lifecycle environmental impacts

LCA is a methodological framework, and as such, it simplifies real phenomena. Not only is each environmental impact associated with ecological processes at a global or regional scale (European Commission 2020b), but all impacts are also dependent on localised processes. Therefore, each LCA analysis is reflective of the background manufacturing conditions assumed and the characterisation factors (CFs) of the impact’s geographical region of reference. According to the CFs for EN ISO 15804:2012 + A2:2019 provided by the European Commission (2023), carbon emissions, ecotoxicity, human toxicity and ionising radiation are assessed based on global CFs, whereas all remaining impacts utilise CFs relevant for the country scale. This distinction agrees with the method used in this analysis, ReCiPe (RIVM 2018). Therefore, (a) geographically dependent CFs and (b) localised manufacturing processes enforce two layers of uniqueness and uncertainty on each LCA study. For example, to investigate the land use impact of 1 solar powered kWh of electricity from the grid, the inventory’s default CF might be used. However, it is unknown whether the grid’s PVs are installed on agricultural, urban or land of any other nature influencing both (a) and (b). Analogous to this, it is unknown if ecotoxicity impacts will end up in water or terrestrial bodies as this will depend on (a). Acknowledging this is important for all impacts, for some because (a) and (b) coincide and for others only because either one exists. It might have been this methodological uncertainty that made EN ISO 15804:2012 + A2:2019 declare abiotic depletion, water depletion, all ecotoxicity metrics and land use as highly uncertain (BS EN 2021a).

The forementioned lead to a new type of performance gap for lifecycle impacts, similar to that associated with building performance. Performance gap has been defined as the difference between the predicted and the actual building performance (Pelsmakers 2015). In a similar fashion, the performance gap of impacts can be defined as ‘the difference in the predicted and the actual environmental impact due to the mismatch between the actual case and the life cycle inventory’.

This performance gap can be tackled by using more granular data. Electricity system operators in the UK and abroad could accompany their projections with spatial-specific assumptions on the nature of land occupied by each generation technology. This can help assess whether land use is going to be a significant impact, as shown here. If, for instance, photovoltaics are to be placed on building rooftops, then land use might not be as significant. Specifying the nature of the land occupied can also help identify whether the land use issue is of the agricultural, urban or other sort. Another way forward might include environmental product declarations (EPDs). EPDs provide more accurate information on the manufacturing of materials and follow EN ISO 15804:2012 + A2:2019. Currently, EPDs do not specify the location of the manufacturing plant or the location of the extraction of raw materials. Such information can help specify land types and emission discharge routes (water, terrestrial or marine bodies). Therefore, more accurate CFs can be used instead of generic ones addressing (a). This could, however, significantly increase the computational complexity of LCAs. Finally, this study calls for considering recycling in module D instead of A so that a conscious effort to minimise upfront impacts takes place.

### Limitations

The results of this paper should be interpreted considering the limitations underpinning the methodology and the very nature of LCA. Overall, limitations can be categorised:

- There is a systemic uncertainty lying in the nature of the assumptions being made. The scenarios used for electricity mix and climate change represent phenomena of a multi-factorial complexity aiming to forecast reality in the deep future. Therefore, a critical uncertainty lies in the analysis that cannot be quantified. Therefore, ‘honesty, humility and transparency are the only sustainable attitudes in the face of this uncertainty’ (Lowe 2006, p. 407).
- The impact of climate change on the LCI’s CFs is not investigated. Doing this would require the implementation of dynamic CFs to see how one unit of impact changes for each climate change scenario. Such datasets are not currently available for the whole multitude of LCA impacts. The ones that exist (e.g. premise) utilise climate-focused reduction parameters and thus are mostly useful for whole life carbon LCAs and surely not for toxicity-related impacts as discussed in the relevant premise publication (Sacchi et al. 2022).
- The manufacturing processes and efficiency remain the same across all future scenarios. Further research can contribute by considering technological improvements of manufacturing processes when it comes to material production.
- Energy performance gap is unavoidable in all dynamic thermal modelling applications. Because of this, the results of the impacts associated with module B6 are such that they represent the assumptions made.
- The windows of the case building are not openable and therefore, no natural ventilation was considered. This led to a considerable increase in cooling consumption when introducing climate change. However, the absence of natural ventilation might overestimate the cooling consumption as opening windows at night could bring significant savings.
- An annual time step was considered for the electricity consumption as future hourly electricity mixes are currently not available in the UK. However, using average annual mixes creates limitations as integrating in situ PV production and heat pumps’ heating induces hourly and seasonal variation.
- Photovoltaics are highlighted as major contributors for some MEP impacts. However, in recent years, their manufacturing processes have considerably improved. Further, monocrystalline silicon is produced in China’s provinces with a high share of hydroelectricity, meaning that using average country mixes for the whole of China might not realistically capture PVs’ fabrication impacts by overestimating their results.
- All impacts are calculated using the default CFs of ReCiPe (Huijbregts et al. 2016). However, recently, concerns are voiced on how the resource use indicators might be underestimated by LCIs. Further, radioactive waste, which is a waste indicator of EN15804, is not investigated. However, building designers can reduce radioactive waste by reducing electricity consumption and generating electricity through renewables.
- All impacts were considered equally important as per ISO 15392:2019 (ISO 2019), treating them all as relevant to buildings. Although this is true for some impacts, it might not be for all. For example, agriculture is responsible for 78% of global eutrophying emissions (Ritchie et al. 2022), leaving little space for building relevance. This has implications on the suggestions to practitioners, and future research can shortlist the environmental impacts when it comes to their relevance to buildings.

## Conclusion

LCA is a methodological framework used to quantify the environmental impacts of buildings. In this paper, the environmental impacts were calculated by considering climate change, grid decarbonisation and electrification of transport.

It was found that electricity mixes influence the results significantly, mainly through module B6. Although embodied modules benefited from grid decarbonisation, this has been very limited. Therefore, reduction of lifecycle impacts cannot rely on future electricity mix evolutions, and efforts should focus on upfront impacts. The electrification of transport had an adverse effect on the embodied IR impact of the case building. Finally, although climate change reduced heating demand, the cooling demand substituted for the savings. However, this is reflective of the case building characteristics, which although has solar protection, does not have night ventilation, and therefore, such a result has to be checked against other assumptions.

Overall, all lifecycle environmental impacts benefit from grid decarbonisation, except for FE, ME and ULO. This highlights that burden shifting might become a concern in future, albeit limited to three impacts. However, the extent of this depends highly on the electricity mix assumed. Sourcing electricity from PVs installed on building rooftops instead of ground-mounted and exporting electricity surplus to the grid might be able to balance these trade-offs.

Finally, a new type of performance gap is proposed for environmental impacts due to (a) geographically dependent CFs and (b) localised manufacturing processes that enforce two layers of uniqueness and uncertainty to each LCA study. This can be defined as ‘the difference between the predicted and the actual environmental impact resulting from the mismatch between the actual case and the life cycle inventory’. The contribution of future research can be twofold. Firstly, the recommendations of the present study can be checked against other results by carrying out sensitivity analyses reflecting other assumptions (e.g. recycling rates, ventilation strategies, thermal properties, shading, orientation and other inventories). Secondly, as embodied impacts did not significantly benefit from grid decarbonisation, future research can consider accounting for technological improvements when evaluating embodied impacts of future processes.

## Data Availability

The data necessary for the dynamic thermal modelling are available, upon reasonable request, from corresponding author Marios Kordilas (marios.kordilas.21@ucl.ac.uk). All other data needed for the analysis are either publicly available or presented in the methodology chapter and supplementary material.

## Appendix

Tables 9, 10, 11, 12.

**Table 9:** Internal heat gains and occupancy profiles

Room	Internal heat gains	Occupancy profile	Temperature setpoint (°C)	Hot Water consumption (l/h.person)	Fresh air (l/s.person)
GF-01	People: 73 Watts X 3 people Lighting: 185 Watts Equipment: -	10:00 - 10:30	21 °C	-	-
GF-02	People: 73 Watts X 6 people Lighting: 280 Watts Equipment: -	12:00 - 14:00	24 - 24 °C	-	-
GF-03	People: 73 Watts X 25 people Lighting: 128 Watts Equipment: -	8:30 - 14:30	23 - 23 °C	-	-
GF-04	People: - Lighting: 100 Watts Equipment: -	12:00 - 14:00	23 - 23 °C	105.9260	-
GF-07	People: 73 Watts X 2 people Lighting: 174 Watts Equipment: 35 Watts	12:00 - 14:00	23 °C	-	-
GF-08	People: 73 Watts X 6 people Lighting: 268 Watts Equipment: 35 Watts	8:30 - 14:30	23 - 23 °C	-	-
GF-09	People: 73 Watts X 2 people Lighting: 171 Watts Equipment: 35 Watts	8:30 - 20:00	23 °C	-	-
GF-10	People: 73 Watts X 1 person Lighting: 340 Watts Equipment: -	12:00 - 14:00	21 °C	-	-
GF-12	People: 73 Watts X 1 person Lighting: 340 Watts Equipment: -	12:00 - 14:00	18 °C	-	-
GF-13	People: 73 Watts X 2 people Lighting: 137 Watts Equipment: 25 Watts	8:00 - 16:00	21 - 23 °C	-	10
GF-14	People: 73 Watts X 6 people Lighting: 229 Watts Equipment: 35 Watts	8:30 - 14:30	23 - 23 °C	-	-
GF-15	People: 208 Watts X 50 people Lighting: 662 Watts Equipment: 25 Watts	7:00 - 16:00	18–18 °C	-	-
GF-16	People: 73 Watts X 12 people Lighting: 45 Watts Equipment: -	12:00 - 14:00	23 °C	105.926	-
GF-17	People: 73 Watts X 25 people Lighting: 88 Watts Equipment: -	8:00 - 16:00	23 °C	-	-
GF-19	People: 73 Watts X 1 people Lighting: 202 Watts Equipment: -	12:00 - 14:00	21 °C	-	-
GF-24	People: 73 Watts X 10 people Lighting: 58 Watts Equipment: -	7:00 - 18:00	23 °C	-	-
GF-25	People: 73 Watts X 5 people Lighting: 50 Watts Equipment: -	7:00 - 18:00	23 °C	-	-
GF-26	People: 73 Watts X 3 people Lighting: 42 Watts Equipment: -	7:00 - 18:00	23 °C	-	-
GF-27	People: - Lighting: 72 Watts Equipment: -	7:00 - 21:00	18 °C	-	-
GF-28	People: - Lighting: 144 Watts Equipment: -	7:00 - 17:00	18 °C	-	-
GF-30	People: 73 Watts X 1 people Lighting: 39 Watts Equipment: -	12:00 - 14:00	21 °C	-	-
GF-31	People: 73 Watts X 38 people Lighting: 351 Watts Equipment: 25 Watts	8:00 - 16:00	21 - 23 °C	-	10
GF-32	People: 73 Watts X 15 people Lighting: 56 Watts Equipment: -	7:00 - 18:00	23 °C	-	-
GF-33	People: 73 Watts X 1 person Lighting: 124 Watts Equipment: -	7:30 - 20:00	21 °C	519.4805	-
GF-34, 45, 41	People: 73 Watts X 3 people Lighting: 32 Watts Equipment: -	7:00 - 19:00	21 °C	-	-
GF-36	People: 73 Watts X 1 person Lighting: 132 Watts Equipment: -	7:30 - 16:00	21 °C	-	-
GF-37	People: 73 Watts X 2 people Lighting: 179 Watts Equipment: 25 Watts	12:00 - 14:00	21 °C	-	-
GF-38	People: 73 Watts X 6 people Lighting: 218 Watts Equipment: 35 Watts	8:30 - 20:00	23 °C	-	-
GF-39	People: 73 Watts X 6 people Lighting: 222 Watts Equipment: 35 Watts	8:30 - 20:00	23 °C	-	-
GF-40	People: 208 Watts X 25 people Lighting: 242 Watts Equipment: 25 Watts	7:00 - 18:00	18 - 18 °C	-	-
GF-42	People: 73 Watts X 15 people Lighting: 65 Watts Equipment: -	8:00 - 16:00	23 °C	-	-
GF-43, 44	People: 73 Watts X 16 people Lighting: 65 Watts Equipment: -	9:00 - 13:00 (weekends)	23 °C	-	-
GF-45, 46, 47, 48, 50	People: 73 Watts X 10 people Lighting: 56 Watts Equipment: -	9:00 - 13:00 (weekends)	23 °C	-	-
GF-49	People: - Lighting: 300 Watts Equipment: -	8:00 - 17:00	18 °C	-	-
IT rooms	People: 73 Watts X 1 person Lighting: 23 Watts Equipment: 1000 Watts	7:30 - 20:00	22 - 22 °C	-	-
FF-01	People: 73 Watts X 2 people Lighting: 229 Watts Equipment: 35 Watts	8:00 - 17:00	21 - 23 °C	0.241	10
FF-02	People: 73 Watts X 2 people Lighting: 228 Watts Equipment: 35 Watts	12:00 - 14:00	21 - 23 °C	0.241	10
FF-03	People: 73 Watts X 2 people Lighting: 211 Watts Equipment: 35 Watts	8:00 - 17:00	21 - 23 °C	0.241	10
FF-04	People: 73 Watts X 2 people Lighting: 213 Watts Equipment: 35 Watts	8:00 - 17:00	21 - 23 °C	0.241	10
FF-05	People: 73 Watts X 12 people Lighting: 376 Watts Equipment: 35 Watts	12:00 - 14:00	21 - 23 °C	0.241	10
FF-06	People: 73 Watts X 10 people Lighting: 562 Watts Equipment: 5 Watts per metre square	8:30 - 9:30 & 12:30 - 14:30	21 - 23 °C	-	-
FF-07	People: 73 Watts X 70 people Lighting: 1477 Watts Equipment: 5 Watts per metre square	7:30 - 14:00	21 - 23 °C	-	-
FF-09	People: 73 Watts X 4 people Lighting: 586 Watts Equipment: 250 Watts per metre square	6:30 - 16:00	18 °C	1.1238	-
FF-12	People: 73 Watts X 1 person Lighting: 319 Watts Equipment: -	12:00 - 14:00	21 °C	-	-
FF-13	People: 73 Watts X 2 people Lighting: 197 Watts Equipment: 25 Watts per metre square	12:00 - 14:00	21 - 23 °C	0.241	10
FF-14	People: 73 Watts X 10 people Lighting: 64 Watts Equipment: -	7:30 - 17:00	23 °C	-	-
FF-15	People: 73 Watts X 4 people Lighting: 268 Watts Equipment: 25 Watts per metre square	8:00 - 17:00	21 - 23 °C	-	-
FF-17	People: 73 Watts X 1 person Lighting: 202 Watts Equipment: -	12:00 - 14:00	21 °C	-	-
FF-19	People: 73 Watts X 8 people Lighting: 263 Watts Equipment: 35 Watts per metre square	7:30 - 17:00	21 - 23 °C	0.2410	10
FF-20	People: 73 Watts X 16 people Lighting: 263 Watts Equipment: 25 Watts per metre square	12:00 - 14:00	21 - 23 °C	-	10
FF-21	People: 73 Watts X 5 people Lighting: 263 Watts Equipment: 25 Watts per metre square	7:30 - 17:00	21 - 23 °C	-	10
FF-22	People: 73 Watts X 1 person Lighting: 263 Watts Equipment: -	12:00 - 14:00	21 °C	-	
FF-23	People: 73 Watts X 40 people Lighting: 1803 Watts Equipment: 20 Watts per metre square	7:30 - 17:00	21 - 23 °C	0.241	10
FF-26	People: 73 Watts X 64 people Lighting: 175 Watts Equipment: -	7:30 - 14:00	21 - 23 °C	-	-
FF-27, 28	People: 73 Watts X 2 people Lighting: 156 Watts Equipment: 25 Watts per metre square	12:00 - 14:00	21 °C	-	-
FF-29	People: 73 Watts X 2 people Lighting: 178 Watts Equipment: 25 Watts per metre square	8:00 - 17:00	21 - 23 °C	0.241	10
FF-30	People: 73 Watts X 2 people Lighting: 215 Watts Equipment: 25 Watts per metre square	8:00 - 19:00	21 - 23 °C	0.241	10
FF-31	People: 73 Watts X 22 people Lighting: 1037 Watts Equipment: 25 Watts per metre square	8:00 - 16:00	21 - 23 °C	0.241	10
FF-32	People: - Lighting: - Equipment: -	7:00 - 17:00	18 °C	-	-
FF-33	People: 73 Watts X 13 people Lighting: 281 Watts Equipment: 25 Watts	8:00 - 18:00	21 - 23 °C	-	10
FF-34	People: 73 Watts X 23 people Lighting: 333 Watts Equipment: 25 Watts	8:00 - 18:00	21 - 23 °C	-	10
FF-35	People: 73 Watts X 18 people Lighting: 333 Watts Equipment: 25 Watts	8:00 - 18:00	21 - 23 °C	-	10
FF-36	People: 73 Watts X 23 people Lighting: 347 Watts Equipment: 25 Watts	8:00 - 18:00	21 - 23 °C	-	10
FF-37	People: - Lighting: 163 Watts Equipment: -	12:00 - 14:00	21 °C	-	-
All remaining unconditioned rooms	People: 73 Watts X 1 person Lighting: - Equipment: -	12:00 - 14:00	-	-	-

[1] All temperature setpoints presented stem from UK NCM (BRE 2022)

[2] People's internal heat gains presented in this Table stem from ASHRAE Handbook of Fundamentals page 18.4 (ASHRAE 2021) and reflect a 73 Watt sensible and a 59 Watt latent load for seated work and a 208 Watt sensible and a 320 Watt latent load for gym areas

[3] Lighting heat gains stem from the case building’s RIBA stage 4 lighting analysis

[4] Equipment loads follow CIBSE Guide A (CIBSE 2015)

[5] Occupancy schedules follow UK NCM (BRE 2022)

[6] Lighting and equipment loads follow the occupancy schedule

[7] Heating and cooling follow the occupancy schedule, being turned on one hour before

[8] Heat pump COP efficiency was set at 3.5

[9] Heating efficiency of FCU was set at 4.5

[10] DHW consumption is taken from UK NCM (BRE 2022)

[11] Where only one temperature setpoint is indicated, no cooling has been considered

[12] Fresh air supply is matched with occupancy schedules

**Table 10:** Electricity consumption of the building’s operational module B6

Climate	Electricity consumption in kWh per meter squared, associated with,				
	Heating	Cooling	DHW	Equipment	Lighting
Current climate	16	3	10	51	8
Medium emissions climate - 2020 s	13	4	10	51	8
High emissions climate - 2020 s	13	4	10	51	8
Medium emissions climate - 2050 s	11	5	10	51	8
High emissions climate - 2050 s	10	6	10	51	8
Medium emissions climate - 2080 s	8	7	10	51	8
High emissions climate - 2080 s	7	9	10	51	8

**Table 11:** Embodied components percentage change (%) compared to Baseline

Percentage change (%) of embodied components compared to Baseline				
	Falling Short	Consumer Transformation	System Transformation	Leading the Way
Climate change	-8%	-8%	-8%	-8%
Ozone depletion	0%	0%	0%	0%
Terrestrial acidification	-9%	-9%	-9%	-9%
Freshwater eutrophication	-1%	-1%	-1%	-1%
Marine eutrophication	-2%	-2%	-2%	-2%
Human toxicity	0%	0%	0%	0%
Photochemical oxidant formation	-5%	-5%	-5%	-5%
Particulate matter formation	-5%	-5%	-5%	-5%
Terrestrial ecotoxicity	0%	0%	0%	0%
Freshwater ecotoxicity	1%	1%	1%	1%
Marine ecotoxicity	1%	1%	1%	1%
Ionising radiation	13%	9%	9%	7%
Agricultural land occupation	0%	0%	0%	0%
Urban land occupation	-1%	-1%	0%	0%
Natural land transformation	-5%	-5%	-5%	-5%
Water depletion	1%	1%	1%	1%
Metal depletion	0%	0%	0%	0%

**Table 12:** Module D percentage change (%) compared to Baseline

Percentage change (%) of module D compared to Baseline				
	Falling Short	Consumer Transformation	System Transformation	Leading the Way
Climate change	0%	0%	0%	0%
Ozone depletion	1%	1%	1%	1%
Terrestrial acidification	0%	0%	0%	0%
Freshwater eutrophication	0%	0%	0%	0%
Marine eutrophication	0%	0%	0%	0%
Human toxicity	0%	0%	0%	0%
Photochemical oxidant formation	0%	0%	0%	0%
Particulate matter formation	0%	0%	0%	0%
Terrestrial ecotoxicity	2%	2%	1%	1%
Freshwater ecotoxicity	0%	0%	0%	-1%
Marine ecotoxicity	0%	0%	0%	0%
Ionising radiation	-1%	4%	4%	6%
Agricultural land occupation	2%	2%	2%	2%
Urban land occupation	0%	0%	0%	0%
Natural land transformation	1%	1%	1%	1%
Water depletion	0%	0%	0%	0%
Metal depletion	0%	0%	0%	0%
